# Evaluation of Total Phenols, Flavonoids, and Antioxidant Activity in the Peel and Flesh of Old Apple Cultivars

**DOI:** 10.3390/foods15183285

**Published:** 2026-09-17

**Authors:** Tamara Edina Gal, Orsolya Borsai, Camelia Tulcan, Roberta Maria Tripon, Florin Adrian Huiban, Ionuț Dascălu, Alin Ionel Dobrei, Olimpia Alina Iordănescu

**Affiliations:** 1 Doctoral School of Engineering of Plant and Animal Resources, Horticulture, University of Life Sciences “King Mihai I” from Timisoara, 119 Aradului Avenue, 300629 Timisoara, Romania; tamara-edina.gal.fita@usvt.ro; 2 Department of Horticulture and Landscape, University of Agricultural Sciences and Veterinary Medicine Cluj-Napoca, 3–5 Mănăștur Street, 400372 Cluj-Napoca, Romania; 3 Department of Silviculture, Faculty of Engineering and Applied Technologies, University of Life Sciences “King Mihai I” from Timisoara, 119 Aradului Avenue, 300629 Timisoara, Romania; cameliatulcan@usvt.ro; 4 Doctoral School of Engineering of Plant and Animal Resources, Biotechnologies, University of Life Sciences “King Mihai I” from Timisoara, 119 Aradului Avenue, 300629 Timisoara, Romania; roberta.tripon@usvt.ro (R.M.T.); florin.huiban@usvt.ro (F.A.H.); 5 Department of Horticulture, Faculty of Engineering and Applied Technologies, University of Life Sciences “King Mihai I” from Timisoara, 119 Aradului Avenue, 300629 Timisoara, Romania; ionut.dascalu@usvt.ro (I.D.); alindobrei@usvt.ro (A.I.D.); olimpiaiordanescu@usvt.ro (O.A.I.)

**Keywords:** *Malus domestica*, DPPH assay, bioactive compounds, polyphenols, antioxidant activity

## Abstract

Apples (*Malus domestica* Borkh.) are widely cultivated due to their storability, adaptability to diverse climatic conditions, versatility, and affordability. In addition to these agronomic advantages, apples are a significant dietary source of nutrients and bioactive compounds, particularly polyphenols, a major class of phytochemicals with several beneficial health effects. The aim of this study was to quantify and compare the total phenolic and flavonoid contents of peel and flesh of eighteen traditional old apple cultivars from the Banat region of Romania, evaluate their antioxidant activity, and characterize the colour parameters and individual phenolic profiles of two selected cultivars. The results showed considerable variation among cultivars, with ‘Caslere’ and ‘Măr de Jupani’ exhibiting the highest contents of bioactive compounds. Across the 18 cultivars, phenolic contents ranged from 218.17–1497.35 µg gallic acid equivalents (GAE)/g dry matter (DM) in the peel and 323.54–1111.65 µg GAE/g DM in the flesh, while flavonoid contents ranged from 1157.43–4796.05 and 1426.01–3697.16 µg rutin equivalents (RE)/g DM, respectively. Antioxidant activity ranged from 33.55–99.51% in the peel and 44.41–88.88% in the flesh, highlighting pronounced tissue-specific differences in bioactive compound distribution. Positive correlation was found between phenolic content and antioxidant activity. High-performance Liquid Chromatography (HPLC) analysis revealed cultivar-specific phenolic profiles, with rosmarinic acid and resveratrol among the predominant compounds, while epicatechin was particularly abundant in the peel of ‘Caslere’ cultivar. Colour analysis further demonstrated distinct differences between the two cultivars, reflecting variation in peel pigmentation and biochemical composition. These findings highlight the nutritional and functional value of traditional apple cultivars and support their conservation and valorization as valuable genetic resources and sources of health-promoting phytochemicals.

## 1. Introduction

Apples (*Malus domestica* Borkh), besides their remarkable storage capacity, wide ecological adaptability [1], and versatility, are a rich source of nutrients and bioactive compounds [2].

Plants naturally produce bioactive compounds (phytochemicals) as part of their secondary metabolism [3], with their biosynthesis often enhanced in response to environmental stressors, such as ultraviolet (UV) radiation, temperature fluctuations, drought, mechanical damage, infections/wounds caused by pathogens, or exposure to toxic metals [4,5,6,7,8]. Phytochemicals include polyphenols, carbohydrates, organic acids, dietary fibers, terpenes, carotenoids [9,10], but polyphenols are the main class found in apples [2]. Currently, more than 60 phenolic compounds are identified in apples, which contribute to plant defense through antioxidant and antimicrobial activities and carry out other functions as well, such as contributing to fruit development, coloration, flavor and storability [11].

The composition and concentration of bioactive compounds in apples, as well as their therapeutic properties, vary significantly among cultivars and is not uniform within the plant. Their qualitative and quantitative profiles differ among anatomical parts, such as the whole fruit, flesh, peel, leaves, seeds, and roots [10], and are also influenced by rootstock, environmental conditions, plant nutritional status, stress factors, fruit maturity, and postharvest storage conditions [12,13,14,15].

Numerous studies have reported higher concentrations of total phenolic compounds in apple peel than in the flesh. This is attributed to the peel’s direct exposure to abiotic and biotic stresses, which determines the intensification of its barrier function and improved adaptation to environmental stressors [7,16,17,18].

Beyond their antioxidant and antimicrobial effects, apple polyphenols have been associated with numerous health-promoting properties, including anti-inflammatory, antidiabetic, cardioprotective, gastroprotective and chemopreventive activities, largely attributed to their ability to reduce oxidative stress and modulate multiple cellular pathways [19,20,21,22,23,24,25,26,27,28,29,30,31,32].

Flavonoids, a major subclass of polyphenols, exhibit antioxidant activity as their principal biological function; however, they also have a broad range of additional bioactivities including anticoagulant, antiviral, antiallergic and anti-inflammatory properties [14,33,34,35,36,37,38].

Fruit enzymatic browning is influenced by the quantity and composition of polyphenols [39], which act as substrates for polyphenol oxidase (PPO) [40].

As cosmopolitan apple cultivars dominate both the global market and the Romanian market, interest in local populations and old varieties has gradually decreased over the years, despite their natural resistance to environmental stressors [17]. While commercial cultivars offer important advantages in terms of productivity, marketability, consistent yield, and uniformity in appearance [41,42], traditional apple cultivars have been shown to contain higher levels of phenolic compounds and greater antioxidant activity compared with modern varieties [17,33,43]. This could be explained by the fact that the emphasis on agronomic and commercial traits during selective breeding of commercial varieties may have contributed to a reduction in the concentration and diversity of certain nutrients and bioactive compounds in their fruits, which ultimately means that they frequently need additional chemicals during processing in order to preserve their flavor and quality [41]. Despite this, commercial apples are still a good source of nutrients. In addition, traditional cultivars’ adaptation to local environmental conditions and inherent resistance/tolerance to pests, diseases and climatic changes may be able to reduce the need for the use of synthetic fertilizers and pesticides, thereby supporting more sustainable production systems [44,45]. Traditional varieties may also provide economic opportunities for small-scale, organic and artisanal producers because their distinctive sensory characteristics and high bioactive compound content can increase the value of processed products [46]. However, lower yield, possible increased variability in fruit size and appearance, and reduced suitability for standardized large-scale marketing may limit the cultivation of traditional apple cultivars.

The health-promoting effects of phytochemicals are largely attributed to their antioxidant activity [2]. The antioxidant activity of apples has been extensively studied and is strongly associated with their total phenolic content [25,47,48,49,50]. However, phenolic compounds are not equally dispersed within the fruits, and their distribution differs significantly between cultivars [51,52,53]. Therefore, this study aimed to investigate differences in the distribution of phenolic and flavonoid compounds between the flesh and peel of eighteen apple cultivars, together with their antioxidant activity. Colorimetric and chromatographic characterization of selected cultivars was also performed to provide further insight into their bioactive profiles. The results provide useful information contributing to the valorization of local germplasm.

## 2. Materials and Methods

### 2.1. Plant Material

**Figure 1:**
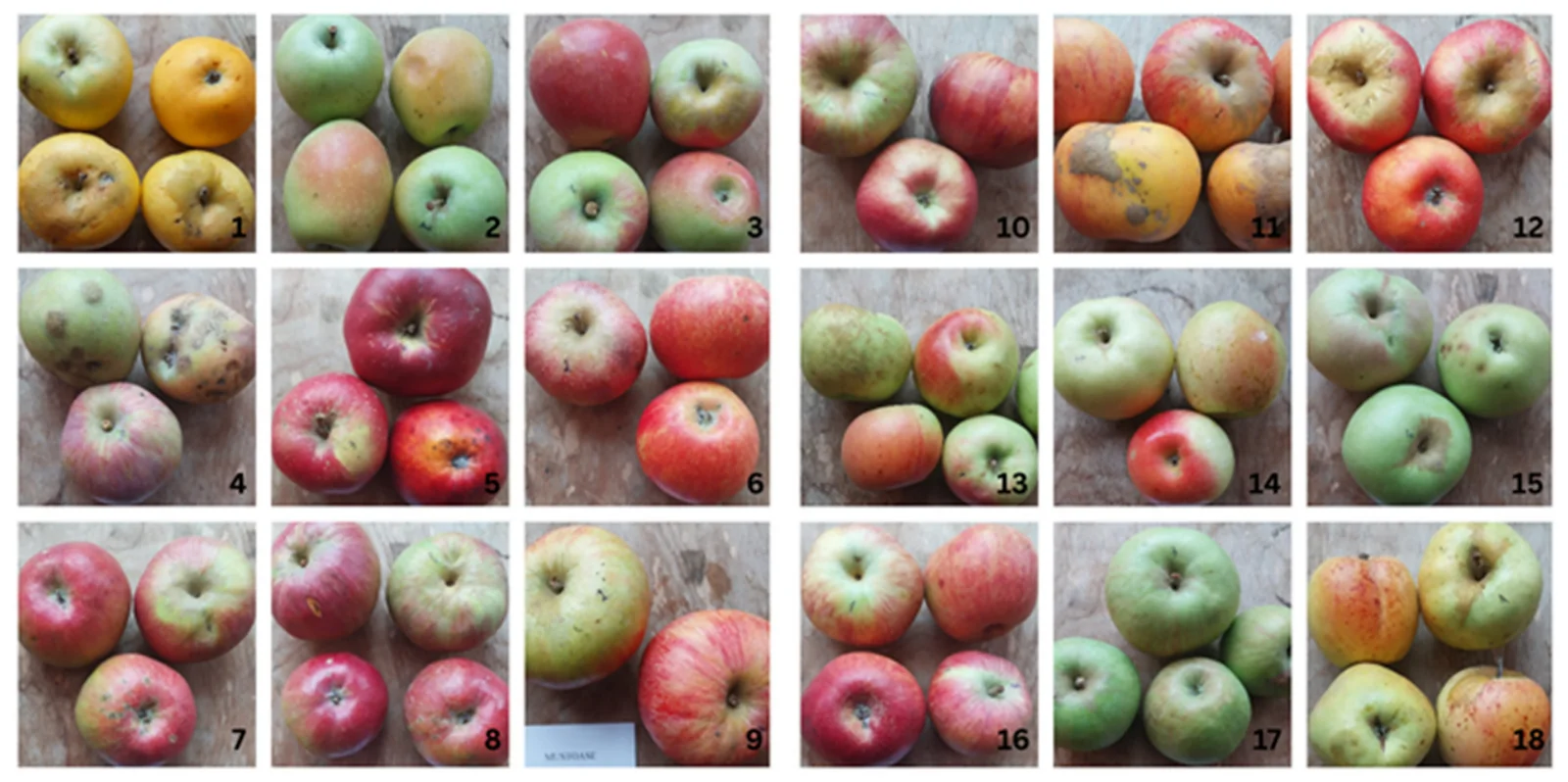
Morphological diversity in the peel appearance of the 18 cultivars: **1**—’Aore’; **2**—’Botu Oii Alb’; **3**—’Botu Oii de Caraș’; **4**—’Carigate’; **5**—’Caslere’; **6**—’Domnesc’; **7**—’Jonathan de munte’; **8**—’Jonathan’; **9**—’Mustoase’; **10**—’Măr de Jupani’; **11**—’Parmen auriu’; **12**—’Pietros’; **13**—’Pogace’; **14**—’Pătul de Vârciorova’; **15**—’Pătul’; **16**—’Șovare’; **17**—’Țigănesc’; **18**—’Vițate’.

For the present study, 18 old apple cultivars were selected: ’Aore’, ’Botu Oii Alb’, ’Botu Oii de Caraș’, ’Carigate’, ’Caslere’, ’Domnesc’, ’Jonathan de munte’, ’Jonathan’, ’Mustoase’, ’Măr de Jupani’, ’Parmen auriu’, ’Pietros’, ’Pogace’, ’Pătul de Vârciorova’, ’Pătul’, ’Șovare’, ’Țigănesc’ and ’Vițate’ (Figure 1). These cultivars are traditional and representative of the Banat region and, following appropriate improvements, could be considered for reintroduction into current assortments.

Scion wood was collected from Banat region germplasm and grafted in 2015 onto M106 rootstock, the primary rootstock utilized in Romania, particularly for the production of commercially viable seedlings [54]. In autumn of 2016, the grafted trees were planted in an experimental orchard in Lugoj (45°41′10″ N, 21°54′2″ E), Timiș county, belonging to the University of Life Sciences “King Mihai I” from Timișoara, at a planting distance of 4 m between the rows and 2 m between the trees within the row.

The apple trees were trained to an improved vase system and subjected to standard phytosanitary treatments (eight sprays per year). Environmental conditions were not controlled. The orchard did not have an irrigation system, and no manual irrigation was carried out during the experimental period.

In September 2023, fruits from multiple trees per cultivar were harvested at horticultural maturity and transported to the laboratory, where they were stored under refrigerated conditions until extract preparation has begun. The number of sampled trees varied among cultivars. A total of 7 trees were sampled for ‘Parmen auriu’; 6 trees for ‘Caslere’; 5 trees for ‘Botu Oii de Caraș’, ‘Jonathan’, ‘Măr de Jupani’ and ‘Pătul de Vârciorova’; 4 trees for ‘Aore’, ‘Botu Oii Alb’, ‘Carigate’, ‘Domnesc’, ‘Mustoase’, ‘Pietros’, ‘Pătul’, ‘Șovare’, ‘Țigănesc’ and ‘Vițate’; 3 trees for ‘Jonathan de munte’ and 2 trees for ‘Pogace’ cultivars. Three fruits were sampled from each tree.

Soluble solid content (SSC) of the fruits was measured using a portable Atago PAL-3780 refractometer (Tokyo, Japan) on approximately 0.3 mL of fresh fruit juice. It varied considerably among the cultivars, ranging from 11.6 to 20.03 ^°^Brix, with ‘Aore’ showing the lowest and ‘Vițate’ the highest value (Table 1).

The: measurable elements of the fruits (diameters, height) were determined with the digital caliper (Insize-1108, Loganville, GA, USA) and the mass of the fruits was determined using the analytical balance (Kern PES620-3M, Balingen, Germany).

### 2.2. Extract Preparation

The apples were manually peeled using a knife, taking care to remove the peel with a consistent thickness of about 1–1.5 mm and to minimize the amount of flesh remaining attached to the peel. Apple peel and flesh samples were lyophilized for 24–48 h using an Ilshin 5512 lyophilizer at −57 °C and 0.05 mTorr. The lyophilized samples were subsequently ground to a fine powder.

For extraction, ethanol with 96% purity (Chimreactiv) was used as a solvent and diluted with distilled water to obtain a concentration of 70% (*v*/*v*).

An aliquot of 2 ± 0.001 g of powdered sample was weighed for each analysis. In order to facilitate the extraction of the compounds from the plant material, 17 mL of ethanol were added. The mixtures were subjected to ultrasound-assisted extraction using an ASonic PRO50 ultrasonic bath (ASONIC d.o.o., Ljubljana, Slovenia) for 30 min at 45 °C with a frequency of 40 kHz and ultrasonic power of 240 W.

Following sonication, the extracts were centrifuged at 3500 rpm for 10 min, at room temperature (22 °C), using a Hettich Universal 320 centrifuge (Andreas Hettich GmbH & Co. KG, Tuttlingen, Germany). The supernatants were collected and adjusted to a final volume of 20 mL with 70% ethanol.

### 2.3. Determination of Total Polyphenol Content

The total polyphenol content of the apple samples was determined by the Folin-Ciocâlteu method, according to the protocol described by Tripon et al. [55].

The final assay volume of 1 mL was obtained by a 1:10 dilution of the initial ethanolic extract with distilled water. An aliquot of 25 μL of the diluted samples was transferred into a 96-well microplate (TPP^®^, TPP Techno Plastic Products AG, Trasadingen, Switzerland), followed by the addition of 125 μL of 10% (*v*/*v*) Folin–Ciocâlteu reagent (WVR Chemicals, Fontenay-sous-Bois, France) diluted with distilled water in a 1:10 ratio, using a multichannel micropipette. Subsequently, 100 μL of 7.5% (*w*/*v*) Na_2_CO_3_ solution were added. The resulting complex was incubated in the dark at room temperature for 30 min.

Optical densities were measured at 760 nm using a Tecan Infinite M1000 Pro spectrophotometer (Tecan Austria GmbH, Grödig, Austria).

The calibration curve was obtained using gallic acid as a standard at eight concentrations (500, 250, 125, 62.5, 31.25, 15.62, 7.81, and 3.9 μg/mL). Based on the calibration curve, a correlation coefficient (R^2^) of 0.9992 was obtained. Total polyphenol concentrations were calculated by interpolation from the calibration curve.

Results were expressed as µg gallic acid equivalents (GAE)/g dry matter (DM) ± standard deviation (SD).

### 2.4. Determination of Flavonoid Content by the Sodium Nitrite Method

The flavonoid content of the apple samples was determined according to the protocol reported by Al-Matani et al. [56], as described below.

The initial ethanolic extracts were diluted 1:4 with distilled water and used for flavonoid content determination. An aliquot of 500 μL of the diluted extract was transferred into a 5 mL volumetric flask. Then, 150 μL of freshly prepared 5% (*w*/*v*) NaNO_2_ solution was added, and the mixture was incubated at room temperature for 5 min. Subsequently, 250 μL of 2% (*w*/*v*) AlCl_3_ solution was added, followed by incubation for an additional 6 min at room temperature, during which a yellow coloration developed. Finally, 250 μL of NaOH solution of 4% (*w*/*v*) was added, and after 10 min at room temperature, a reddish-brown complex was formed.

After addition of the reagents, the reaction mixture was adjusted to a final volume of 5 mL with distilled water. Absorbance was measured at 510 nm using a PerkinElmer Lambda 25 UV-VIS spectrophotometer (PerkinElmer, Singapore).

Rutin (1 mg/mL) was used as a reference standard to prepare six calibration solutions at 0, 62.5, 125, 250, 500 and 1000 μg/mL. Based on the calibration curve, a correlation coefficient (R^2^) of 0.9980 was obtained, and flavonoid concentrations in the samples were calculated by interpolation from the curve.

Results were expressed as µg rutin equivalents (RE)/g DM ± SD.

### 2.5. Evaluation of Total Antioxidant Activity via DPPH Assay

The half-maximal inhibitory concentration (IC_50_) of the extracts was determined using the 2,2-diphenyl-1-picrylhydrazyl (DPPH) radical-scavenging assay. IC_50_ represents the extract concentration required to reduce the initial DPPH radical concentration by 50%. A lower IC_50_ value indicates a higher radical scavenging activity. The percentage of inhibition was calculated to obtain the proportion of free radicals neutralized by the sample.

The assay was based on absorbance measurements at 515 nm, the maximum wavelength for DPPH. In the presence of antioxidants, the absorbance of DPPH decreases proportionally to the radical scavenging activity, accompanied by a color change from violet to yellow.

The antioxidant activity of the extracts was evaluated using the DPPH assay, following the protocol described by [57]. A 2.5 × 10^−4^ MolL^−1^ DPPH solution was prepared in methanol.

In order to obtain five concentrations of each extract, serial dilutions were performed in 70% ethanol, as described in Table 2.

From each extract concentration, 10 µL were transferred into a 96-well microplate (TPP^®^, TPP Techno Plastic Products AG, Trasadingen, Switzerland). Using a multichannel pipette, 200 µL of the DPPH reagent were added to each well. The reaction mixtures were incubated in the dark, and absorbances were measured at 515 nm after 10, 15 and 30 min.

IC_50_ values and percent inhibitions (%) for the five extract concentrations were calculated using the Hill equation [58] in GraphPad Prism 10.4.1. (GraphPad Software, LLC, Boston, MA, USA):

$$Y=Min+\frac{Max-Min}{1+{\left(\frac{X}{IC_{50}}\right)}^{Hill\;coefficient}}$$

In the equation, Y represents the percentage of DPPH radical-scavenging activity at concentration X. X denotes the concentration of the tested sample, while Max and Min represent the maximum and minimum asymptotic response values. The Hill slope coefficients calculated for the tested samples ranged from 0.12 to 0.86. IC_50_ values were expressed in mg/mL, calculated based on the correlation between the extract concentrations and the corresponding amount of fruit sample used for each extract.

### 2.6. High-Performance Liquid Chromatography (HPLC) Analysis of Individual Polyphenolic Compounds for ‘Măr De Jupani’ and ‘Caslere’ Cultivars

Individual polyphenolic compounds were identified and quantified using an HPLC system (Shimadzu 2010 EV, Shimadzu, Kyoto, Japan) equipped with an SPD-10A UV detector and an EC 150/2 NUCLEODUR C18 Gravity SB analytical column (150 × 2 mm, 5 μm; Macherey-Nagel GmbH & Co. KG, Dueren, Germany).

The chromatographic separation was performed using a binary mobile phase consisting of acidified water (solvent A, pH 3 adjusted with formic acid) and acidified acetonitrile (solvent B, pH 3 adjusted with formic acid). Elution was carried out under gradient conditions as follows: 5% B from 0.01 to 20 min, a linear increase from 5% to 40% B between 20.01 and 50 min, followed by an increase from 40% to 95% B from 50 to 55 min, with 95% B maintained until 60 min.

The flow rate was set at 0.2 mL/min, while the column temperature was maintained at 20 °C throughout the analysis. Detection was performed at 280 and 320 nm, and quantification was achieved using external calibration curves prepared within the concentration range of 20–50 μg/mL, according to the method previously described by Dossa et al. [59].

### 2.7. Colour Analysis for ‘Măr De Jupani’ and ‘Caslere’ Cultivars

Fruit peel and flesh colour parameters were determined using a Konica Minolta CM-5 spectrophotometer (Konica Minolta, Osaka, Japan). Colour was measured in the CIELAB Colour Measuring System using the Specular Component Excluded (SCE) mode with a 30 mm measurement aperture, D65 illuminant, and 10° standard observer. The results were expressed with L* (lightness), a* (green to red axis, with +a* indicating red, and −a* indicating green), and b* (blue to yellow axis, with +b* indicating yellow, and −b* indicating blue) [60]. Measurements were performed using the instrument’s Manual Average Measurement mode.

### 2.8. Statistical Analysis

Statistical analyses were performed using IBM SPSS Statistics version 19.0 (IBM Corp., Armonk, NY, USA). All measurements were performed in triplicate, and the results are expressed as mean ± standard deviation. Descriptive statistics were calculated for the morphologic, biochemical, and antioxidant parameters.

Differences in total phenolic content, total flavonoid content, and antioxidant activity parameters (IC_50_ and percentage inhibition) among the investigated cultivars and between fruit tissues (flesh and peel) were evaluated using one-way analysis of variance (ANOVA), at 95% confidence level. Further, Tukey’s multiple range test was performed at *p* < 0.05 to determine significant differences between the means.

Pearson’s correlation analysis was performed to assess the linear relationships between morphological characters, total phenolic content, total flavonoid content, and antioxidant activity parameters (IC_50_ and percentage inhibition).

Principal component analysis (PCA), correspondence analysis (CA) and hierarchical cluster analysis (HCA), along with a heatmap, were also performed.

## 3. Results

### 3.1. Morphological and Biochemical Characterization of the Cultivars

#### 3.1.1. Morphological Characterization

The morphological characterization of the analyzed apple cultivars, based on fruit diameter, height, and mass, is presented in Table 3. These parameters provided an initial characterization of the studied genotypes and highlighted the diversity of fruit morphology within the analyzed apple collection.

Additionally, fruit shape index (FSI) was calculated as the ratio of mean fruit height to mean large diameter (fruit width). An FSI value greater than 1 indicates an elongated fruit shape, a value equal to 1 indicates a round shape, and values below 1 indicate a flattened or oblate shape [61]. Based on these criteria, the fruits of the 18 cultivars can be classified into two categories: ‘Botu Oii Alb’ exhibited approximately round fruits (FSI = 1.06), whereas the other cultivars exhibited more or less flattened fruits, with FSI values below 1 and relatively close to round shape.

#### 3.1.2. Biochemical Characterization

- Total phenolic content (TPC)

**Figure 2:**
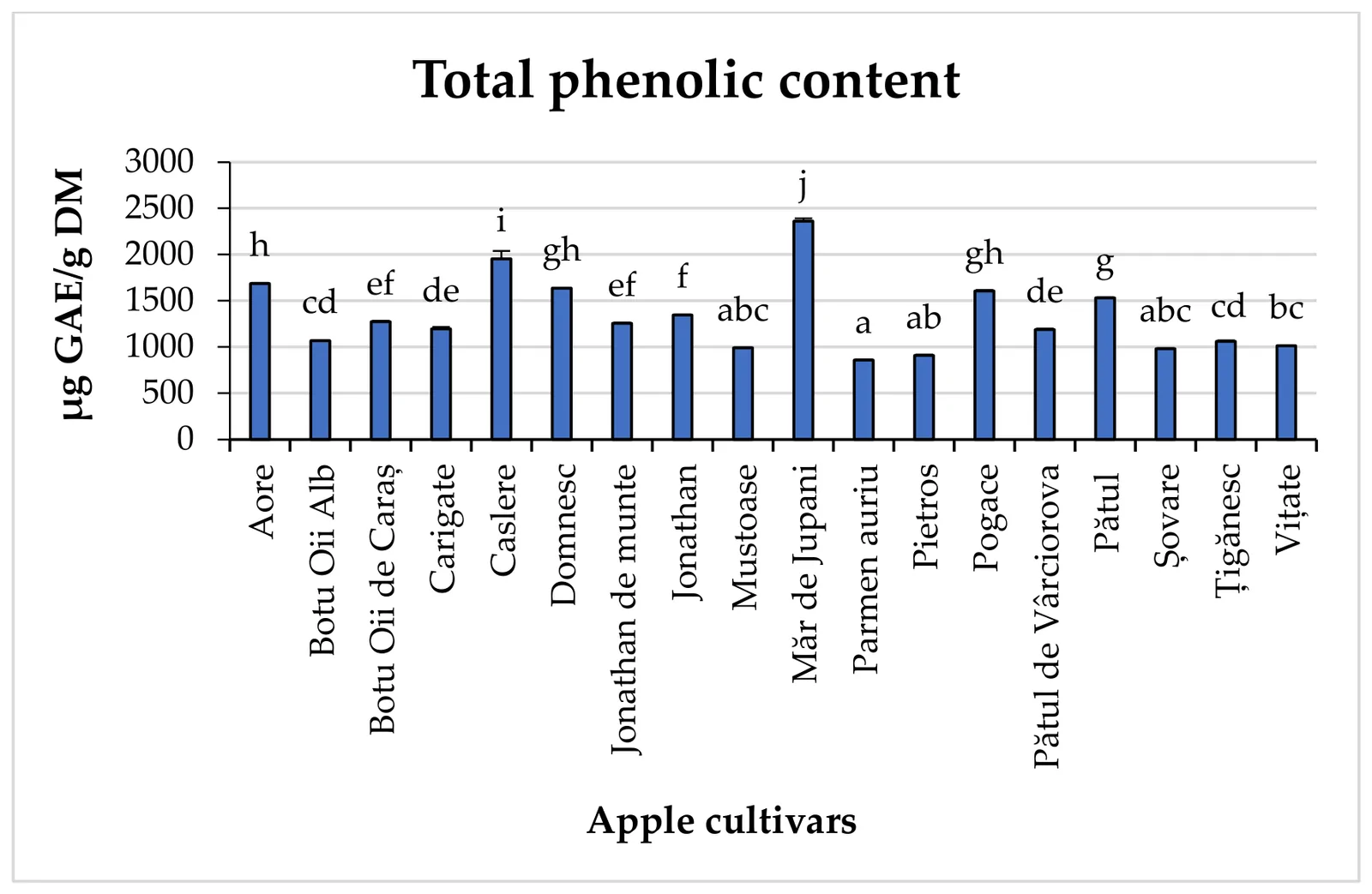
Total phenolic content of the investigated apple cultivars. The data presented are means ± standard deviation (SD). Different lowercase letters above the bars indicate statistically significant differences between the means, according to Tukey’s HSD test at *p* < 0.05 (n = 3).

The total phenolic content varied significantly among the 18 apple cultivars, ranging from below 1000 to over 2300 µg gallic acid equivalents (GAE)/g dry matter (DM) (Figure 2). The highest values were recorded in ‘Măr de Jupani’ (2361.18 ± 31.86 µg GAE/g DM), ‘Caslere’ (1953.57 ± 86.39 µg GAE/g DM) and ‘Aore’ (1686.40 ± 9.36 µg GAE/g DM), which showed significantly higher phenolic levels than the other cultivars. The results are in accordance with previous reports [7,8,17,18,62,63,64,65], supporting the observation that apple peel generally contains higher polyphenol levels than the flesh.

Marked differences in phenolic distribution between peel and flesh were observed among cultivars (Table 4), allowing their classification into high-, mid-, and low-phenolic groups.

Within the high-phenolic group, ‘Măr de Jupani’ exhibited the highest TPC, with 63.42% of the phenolics localized in the peel. A similar distribution was observed in ‘Caslere’, which showed a markedly higher phenolic proportion in the peel (75.48%), as well as ‘Pogace’ (72.70%). In contrast, ‘Aore’ displayed a distinct distribution pattern, with the flesh contributing 65.91% of the total phenolic content (1111.65 ± 16.03 µg GAE/g DM).

Among the mid-range phenolic cultivars, total phenolic content varied between 1256.07 ± 8.06 and 1635.26 ± 7.41 µg GAE/g DM. Although both tissues contributed to the total phenolic content, the peel remained the predominant source, accounting for approximately 54.12–71.17% of total phenolics.

The low-phenolic group included ‘Parmen auriu’, ‘Pietros’, ‘Șovare’, ‘Țigănesc’ and ’Vițate’, with TPC values ranging from 857.87 ± 7.21 to 1061.89 ± 10.11 µg GAE/g DM. In most of these cultivars, phenolics were predominantly localized in the peel (62.26–64.61%). The only exception was ’Pietros’, in which the flesh accounted for 75.96% of the total phenolic content.

Overall, the results indicate that apple peel represents the primary reservoir of phenolic compounds in most cultivars, generally containing two- to three-fold higher concentrations than the flesh. The high TPC observed in cultivars such as ‘Măr de Jupani’, ‘Caslere’ and ‘Pogace’ was primarily associated with their phenolic-rich peel, whereas ‘Aore’ and ‘Pietros ’ were distinguished by their unusually high flesh phenolic content (the flesh of ‘Aore’ cultivar accounts for 65.91% of the total phenolic content of the fruits, while the flesh of ‘Pietros’ contributes in an even higher proportion of 75.96% to the total phenolic content). The higher phenolic content observed in the flesh of ‘Aore’ and ‘Pietros’ may be related to cultivar-dependent genetic differences affecting the biosynthesis and accumulation of phenolic compounds. Phenylpropanoid and flavonoid biosynthesis pathways play central roles in phenolic metabolism, and previous genetic research has demonstrated a substantial genetic component underlying variation in phenolic concentration [66]. In particular, on linkage group 16, a mQTL hotspot linked to several phenolic compounds in apple flesh was found; leucoanthocyanidin reductase 1 (MdLAR1) was suggested as a strong candidate gene underlying this region. Further investigation revealed that variations in MdLAR1 expression were associated with differences in procyanidin accumulation, supporting a link between genetic variation, regulation of phenolic biosynthesis, and the final concentration of phenolic metabolites. Although these studies did not specifically investigate ‘Aore’ and ‘Pietros’, they suggest that their higher flesh phenolic content may reflect genotype-dependent regulation of phenolic biosynthesis, potentially involving genes such as MdLAR1, rather than differences in phenolic distribution alone.

These findings emphasize the nutritional importance of apple peel consumption, as it substantially enhances polyphenol intake and, for certain cultivars, may increase antioxidant intake by more than 50%.

- Total flavonoid content (TFC)

**Figure 3:**
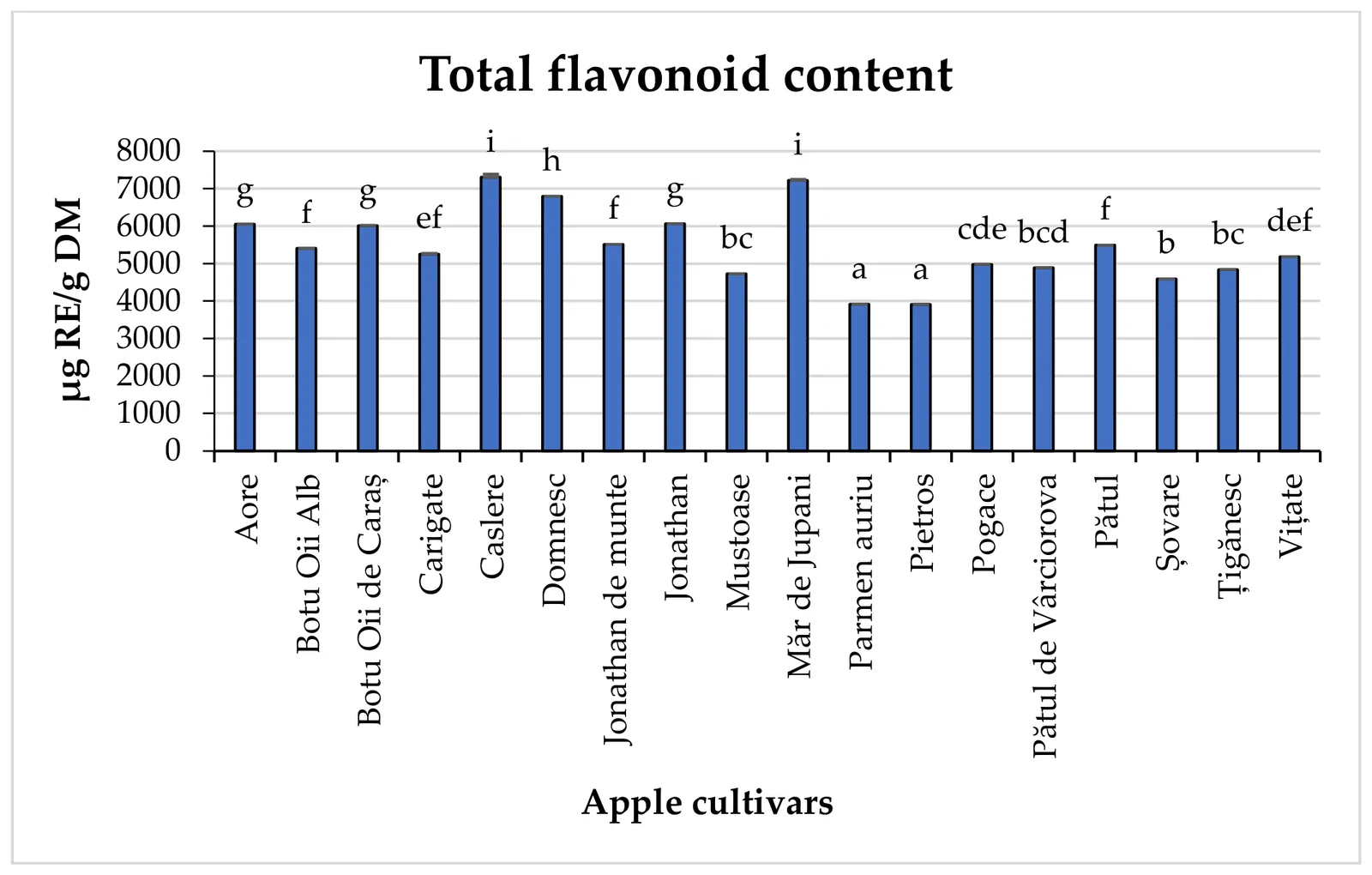
Total flavonoid content of the investigated apple cultivars. The data presented are means ± SD. Different lowercase letters above the bars indicate statistically significant differences between the means, according to Tukey’s HSD test at *p* < 0.05 (n = 3).

Flavonoids represent one of the most significant classes of natural phenolic compounds with diverse biological activities [67]. Analysis of TFC revealed significant variation among the 18 apple cultivars, ranging from 3908.30 ± 10.79 to 7306.2 ± 86.29 µg rutin equivalents (RE)/g DM (Figure 3). Cultivars such as ‘Caslere’ (7306.2 ± 86.29 µg RE/g DM) and ‘Măr de Jupani’ (7220.01 ± 31.86 µg RE/g DM) exhibited particularly high flavonoid concentrations, whereas the lowest values were recorded in ‘Pietros’ (3908.30 ± 10.79 µg RE/g DM) and ‘Parmen auriu’ (3914.27 ± 7.31 µg RE/g DM).

Based on TFC values, the cultivars were classified into three groups: low-flavonoid group (3908–5000 µg RE/g DM), comprising seven cultivars (38.88%); medium-flavonoid group (5001–6000 µg RE/g DM), comprising five cultivars (27.77%); and high-flavonoid group (>6000 µg RE/g DM), consisting of six cultivars (33.33%). The lowest values were recorded in ‘Pietros’ (3908.30 ± 10.79 µg RE/g DM) and ‘Parmen auriu’ (3914.27 ± 7.31 µg RE/g DM).

Differences in flavonoid distribution between peel and flesh were observed across cultivars (Table 5). Except for ‘Aore’, ‘Măr de Jupani’, and ‘Pietros’, all cultivars displayed markedly higher flavonoid concentrations in the peel, with values approximately 1.8-fold greater than those measured in the flesh. This distribution pattern agrees with previous studies reporting considerably higher flavonoid concentrations in apple peel compared with flesh [53,68].

Several cultivars showed distinct tissue-specific accumulation patterns. ’Mustoase’ and ’Măr de Jupani’ exhibited a relatively balanced distribution of flavonoids between flesh and peel, with similar concentrations in both tissues. In contrast, ’Pietros’ displayed a flesh-dominant profile, characterized by comparatively low flavonoid levels in the peel, consistent with its phenolic distribution pattern observed for TPC.

Cultivars with high TFC, including ‘Caslere’ (peel: 4796.05 ± 0.37; flesh: 2510.14 ± 1.37 µg RE/g DM), ‘Parmen auriu’ (peel: 2488.25 ± 32.05; flesh: 1426.01 ± 0.61 µg RE/g DM) and ‘Domnesc’ (peel: 3859.64 ± 6.90; flesh: 2932.67 ± 6.96 µg RE/g DM), exhibited elevated flavonoid concentrations in the peel and moderate-to-high levels in the flesh. Overall, these findings confirm that apple peel represents the major flavonoid source in most cultivars, and its removal may reduce flavonoid intake by more than 2-fold in case of some cultivars.

- Antioxidant activity

The antioxidant activity of apples is primarily attributed to the presence of phenolic compounds in the fruits, mainly due to their ability to scavenge free radicals and reduce oxidative damage [69,70,71]. In the present study, antioxidant activity was evaluated in both peel and flesh extracts from the 18 apple cultivars using half-maximal inhibitory concentration (IC_50_) and percentage inhibition values (the efficiency of a sample in neutralizing reactive free radicals). IC_50_ values represent the concentration required to inhibit 50% of the target radical species; therefore, lower IC_50_ values indicate stronger antioxidant activity. The results are presented as mean values in Table 6.

Substantial variation in antioxidant capacity was observed among cultivars and tissues. In general, peel extracts exhibited lower IC_50_ values and higher inhibition percentages than flesh extracts, indicating greater antioxidant potential (several peel samples showed inhibition values above 99%). The strongest antioxidant activity was observed in ‘Caslere’ peel, as reflected by the lowest IC_50_ value (1.11 ± 0.0020 mg/mL) and the highest percentage inhibition (99.51%), followed by ‘Măr de Jupani’ (IC_50_ = 1.19 ± 0.0039 mg/mL; 99.31% inhibition) and ‘Pogace’ (IC_50_ = 1.43 ± 0.0022 mg/mL; 88.19% inhibition).

Among flesh samples, ‘Aore’ showed the highest antioxidant activity (IC_50_ = 1.93 ± 0.0262 mg/mL; 88.86% inhibition), followed by ‘Domnesc’, ‘Pietros’, ‘Botu Oii de Caraș’, and ‘Vițate’, which exhibited IC_50_ values between 3.00 and 3.90 mg/mL. In contrast, the weakest antioxidant activity was recorded in ‘Parmen auriu’ flesh (IC_50_ = 7.42 ± 0.0246 mg/mL; 44.41% inhibition) and ‘Jonathan’ flesh (IC_50_ = 6.61 ± 0.00 mg/mL; 55.56% inhibition).

Although peel generally exhibited higher antioxidant activity, two cultivars showed atypical patterns. ‘Pietros’ peel displayed the highest IC_50_ value among peel samples (7.22 ± 0.0021 mg/mL), corresponding to a low inhibition percentage (33.55%), while ‘Aore’ showed greater antioxidant activity in the flesh than in the peel.

Overall, except for ‘Aore’ and ‘Pietros’, all cultivars showed lower IC_50_ values in peel compared with flesh. The mean IC_50_ value was 2.74 mg/mL for peel extracts and 4.73 mg/mL for flesh extracts, indicating a 1.73-fold lower IC_50_ and consequently greater antioxidant capacity in the peel. These findings are consistent with previous reports demonstrating the greater antioxidant potential of apple peel compared with flesh [18,64,71,72,73].

- Statistical analysis

Correlation analysis of phenolic compounds and antioxidant activity.

**Figure 4:**
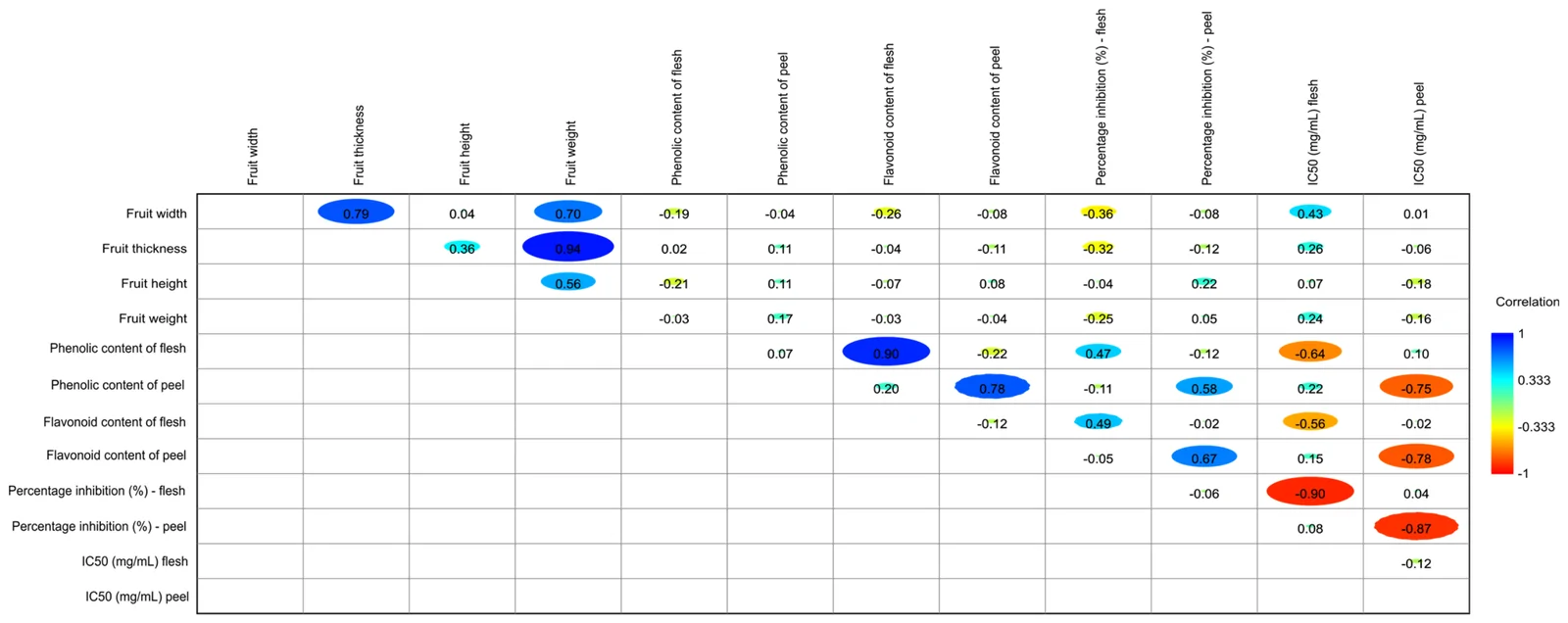
Pearson correlation coefficients (‘*r*’ value) between the analyzed parameters of the 18 apple cultivars. Positive correlations are displayed in dark and light blue colours while negative correlations in yellow and red. The intensity of the colour and the size of the ellipses are directly proportional to the correlation coefficients. All values greater than 0.33 indicate statistically significant values of ‘*r*’ at *p* < 0.05.

Pearson’s correlation analysis revealed several positive and negative relationships among the biochemical parameters evaluated in the peel and flesh of the old apple cultivars (Figure 4). These results demonstrate further that the antioxidant properties of the investigated old apple cultivars resulted from complex relationships among the different groups of bioactive compounds and their tissue-specific distribution.

The strong positive correlations observed among several phenolic and antioxidant parameters (r = 0.94 and r = 0.90) support the important contribution of phenolic compounds and flavonoids to antioxidant activity, whereas the negative relationships (r = −0.56, −0.64, −0.75, −0.78, −0.87, and −0.90) indicate that the biochemical composition of apple peel and flesh is not governed by a uniform accumulation pattern. From a biochemical point of view, such relationships are expected because phenolic compounds constitute an important component of the antioxidant system of apple fruit. Both total phenols and flavonoids include compounds capable of donating electrons or hydrogen atoms and stabilizing reactive species.

Consequently, cultivars characterized by greater accumulation of these compounds may also exhibit greater antioxidant activity. However, correlations below unity indicate that antioxidant capacity cannot be attributed exclusively to total phenolic or flavonoid concentrations. Differences in the phenolic profile, relative abundance of individual compounds, their chemical structure, and the presence of other antioxidant constituents may also contribute to the antioxidant response.

These findings further highlight the considerable biochemical diversity present among old apple cultivars and their potential value as sources of health-related phytochemicals.

##### Multivariate Analysis

**Figure 5:**
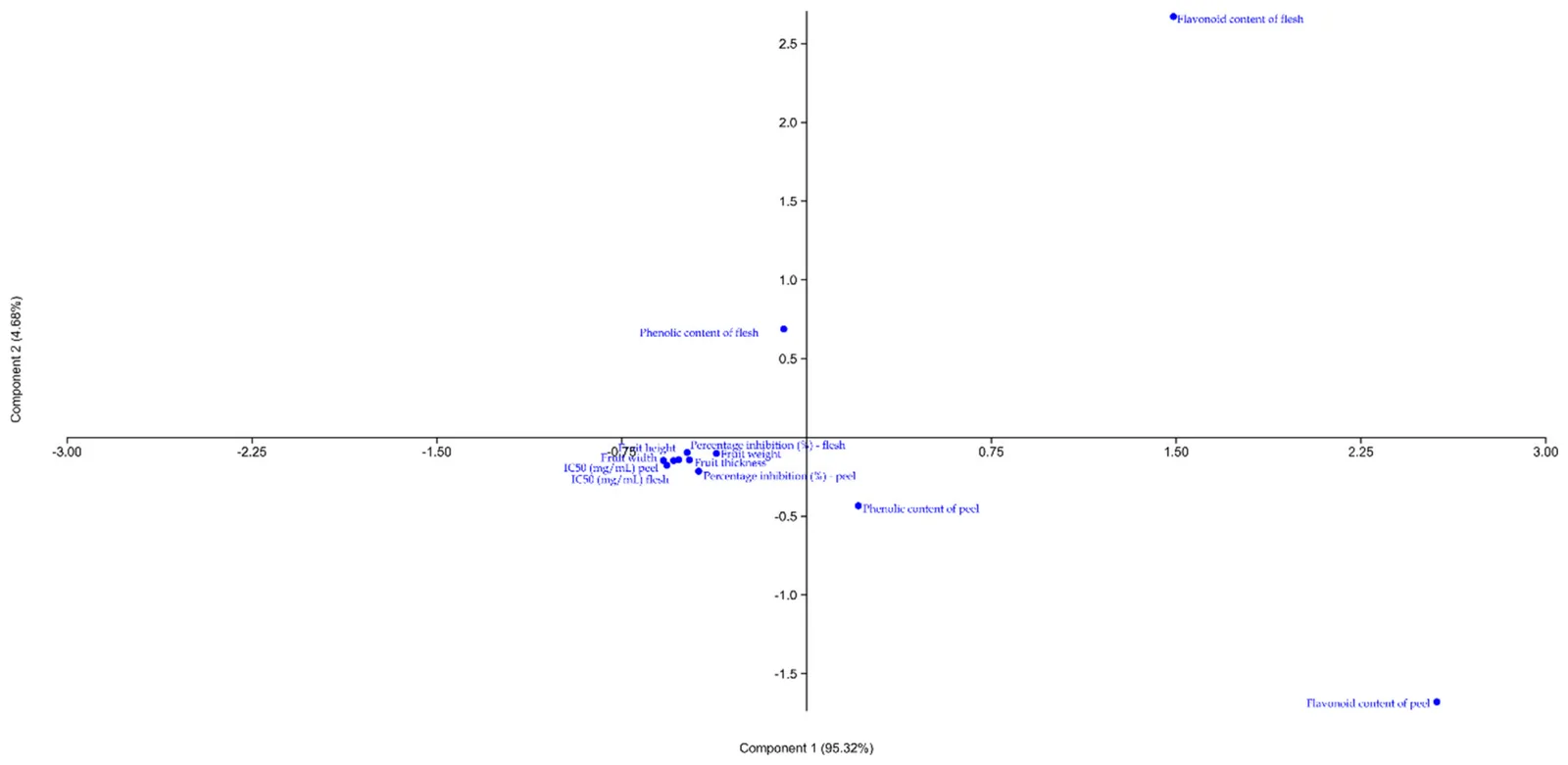
Principal component analysis (PCA) plot: the multivariate analysis performed for 18 apple cultivars based on the analyzed parameters.

Principal Component Analysis (PCA) revealed a clear differentiation among the biochemical characteristics of the old apple cultivars (Figure 5). Most of the evaluated variables were grouped relatively close to the center of the PCA, indicating a generally similar contribution to the overall variability. The morphological parameters, including fruit weight and height, were positioned close to antioxidant activity (DPPH) and several phenolic and flavonoid variables, suggesting associations among these characteristics.

In contrast, some biochemical parameters were clearly separated from the central group. Flavonoid content of the flesh was positioned in the upper part of PCA, whereas flavonoid content of the peel was located in the opposite, lower-right region, indicating markedly different patterns of variation between the two fruit tissues. Phenolic content of the flesh was also separated from the main cluster, while phenolic content of the peel occupied a more distinct position toward the right side of the central group. This distribution confirms that the accumulation of phenolic compounds and flavonoids in apple peel and flesh does not follow an identical pattern across cultivars.

Overall, PCA highlights a pronounced tissue-specific differentiation of bioactive compounds, particularly for flavonoids, while most morphological and antioxidant-related variables formed a comparatively compact group. Together with the correlation analysis, these results indicate considerable biochemical diversity among the old apple cultivars and support the separate evaluation of peel and flesh when assessing their phenolic composition and antioxidant potential.

##### Correspondence Analysis

**Figure 6:**
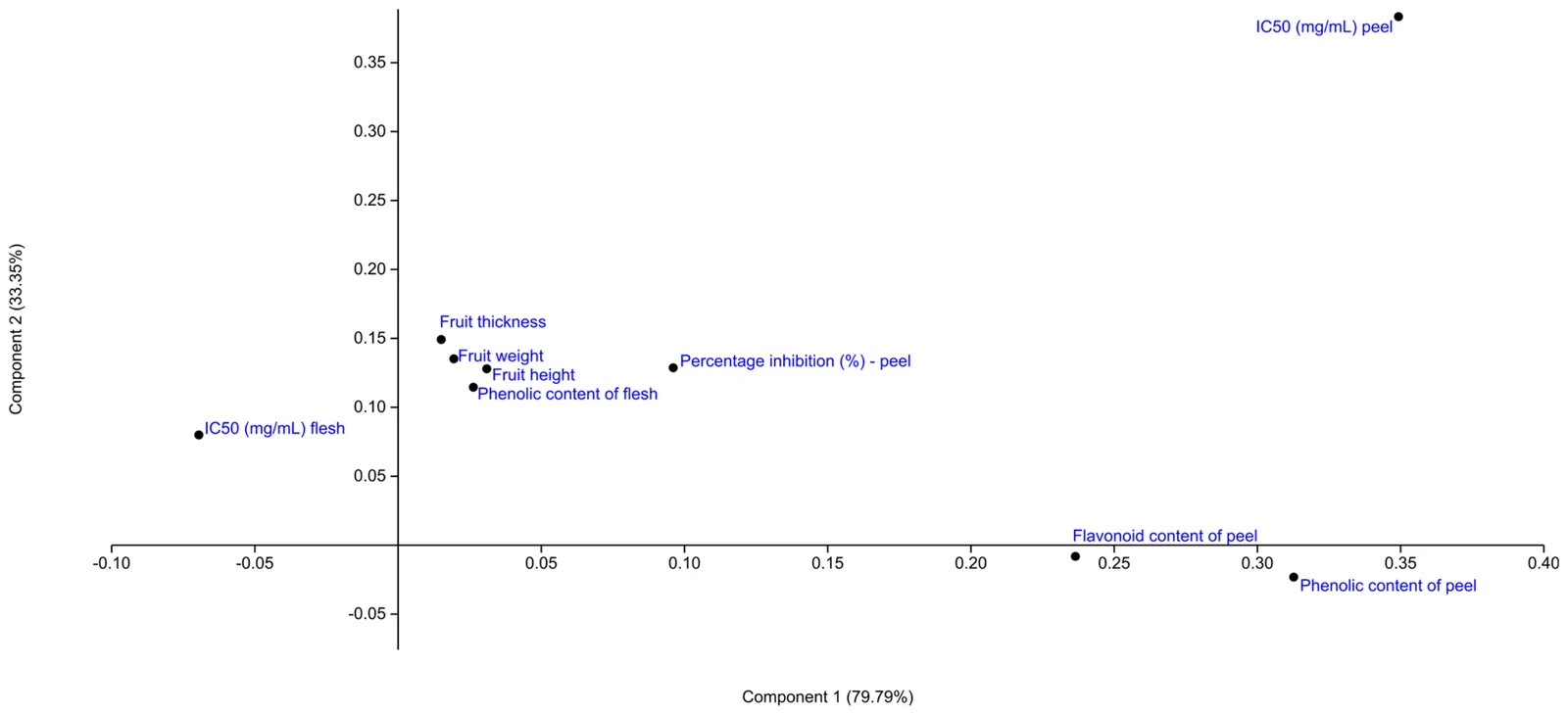
The correspondence analysis (CA) of the parameters analyzed in the 18 apple cultivars.

Correspondence analysis (CA) revealed a clear separation of the morphological and biochemical characteristics evaluated in the old apple cultivars (Figure 6). Fruit thickness, fruit weight, fruit height, and phenolic content of the flesh were closely grouped, indicating a similar pattern of variation among the cultivars. The IC_50_ value of the flesh was also located relatively close to this group, whereas percentage inhibition in the peel occupied a more distinct position.

The characteristics associated with the peel showed the greatest differentiation. Phenolic content and flavonoid content of the peel were positioned in the lower-right part of the ordination and were separated from the cluster formed by fruit morphological traits and phenolic content of the flesh. In contrast, the IC_50_ value of the peel was the most distant characteristic, occupying the upper-right region of the plot. Its separation from peel phenolic and flavonoid contents indicates an inverse pattern between IC_50_ and the accumulation of these bioactive compounds. This is biologically meaningful because a lower IC_50_ corresponds to stronger antioxidant activity; therefore, increased phenolic and flavonoid concentrations would be expected to be associated with lower IC_50_ values. Thus, CA indicates a clear tissue-dependent pattern in the biochemical characteristics of the fruit, with peel-related phenolic and antioxidant parameters showing greater differentiation than those of the flesh. The proximity of fruit size traits and flesh phenolic content suggests that these variables exhibited comparatively similar patterns across the investigated cultivars, whereas the marked separation among peel phenolics, flavonoids, and IC_50_ highlights the distinctive contribution of the peel to the biochemical diversity and antioxidant properties of old apple cultivars.

##### Hierarchical Cluster Analysis

**Figure 7:**
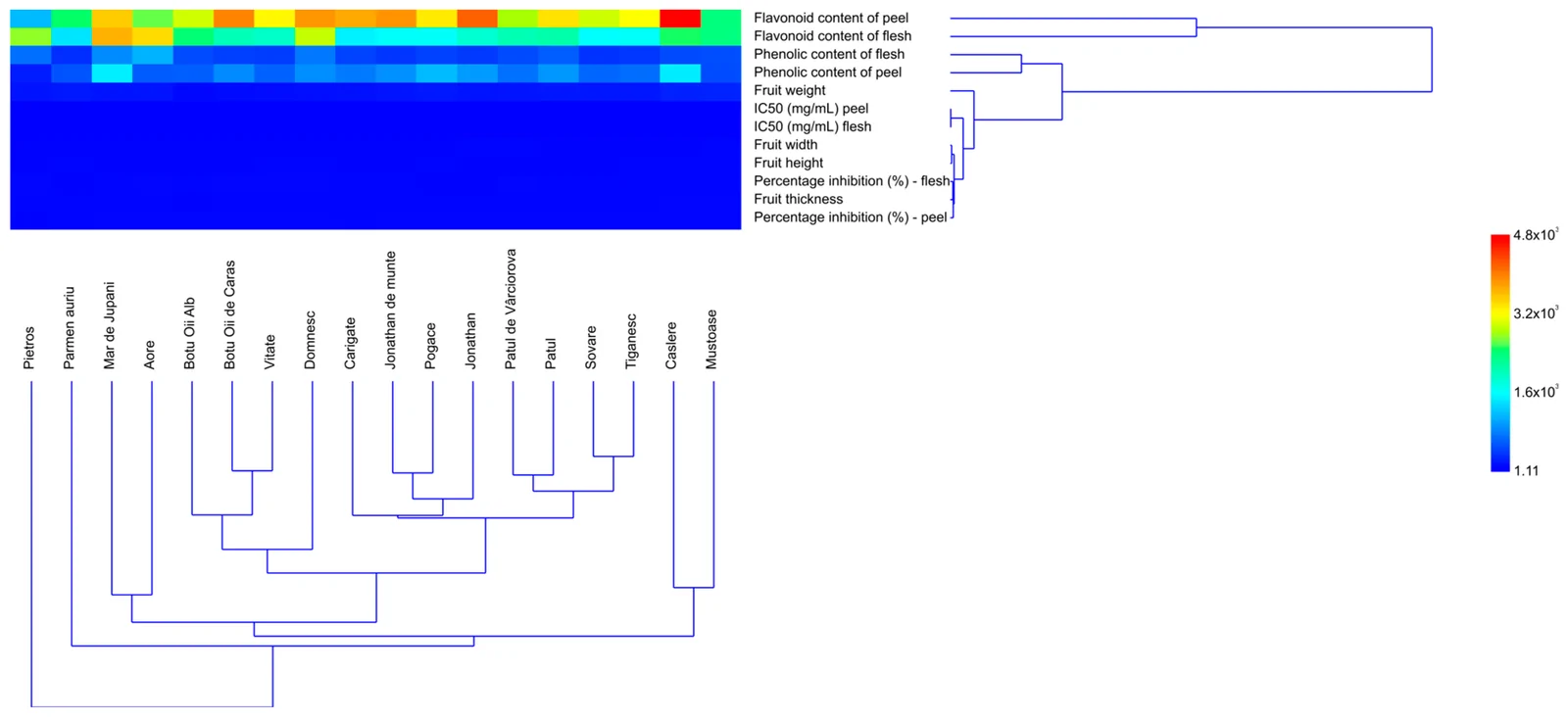
Hierarchical cluster analysis (HCA) and heatmap of morphological characteristics, phenolic and flavonoid contents, and antioxidant activity in the peel and flesh of old apple cultivars. Similarities among cultivars and analyzed traits are represented by the corresponding column and row dendrograms, respectively.

Hierarchical cluster analysis (HCA) revealed considerable diversity among the old apple cultivars based on the combined morphological and biochemical characteristics (Figure 7). The cultivar dendrogram showed several distinct groups and subgroups, indicating that the investigated genotypes differed substantially in their overall fruit characteristics and in the distribution of bioactive compounds between peel and flesh.

Several cultivars showed relatively close associations. ‘Vițate’ and ‘Domnesc’ were grouped within the same small subcluster, indicating similar overall profiles for the analyzed characteristics. Likewise, ‘Jonathan de munte’ and ‘Pogace’ were grouped together and subsequently associated with ‘Jonathan’, suggesting similarities among these cultivars in their combined morphological and biochemical characteristics.

Another distinct part of the dendrogram included ‘Pătul de Vâlciorova’ and ‘Pătul’, which formed a close pair, while ‘Șovare’ and ‘Țigănesc’ constituted another clearly defined subcluster. These two pairs subsequently joined within a larger cluster, indicating a greater degree of similarity to each other than to cultivars located on more distant branches. ‘Carigate’ was also associated with the central group, although at a greater linkage distance.

Among the remaining cultivars, ‘Aore’ and ‘Măr de Jupani’ were relatively closely associated and subsequently grouped with the larger central cluster. In contrast, ‘Pietros’ and ‘Parmen auriu’ occupied more isolated positions and joined the other cultivars only at relatively large linkage distances, indicating more distinctive overall profiles. On the opposite side of the dendrogram, ‘Caslere’ and ‘Mustoase’ formed a separate pair that was also relatively distant from the main central group.

Overall, the clustering pattern demonstrates substantial phenotypic and biochemical diversity among the investigated old apple cultivars. The close grouping of cultivars such as ‘Vițate’–‘Botu Oii Alb’, ‘Jonathan de munte’–‘Pogace’, ‘Pătul de Vâlcirova’–‘Pătul’, and ‘Șovare’–‘Țigănesc’ reflects similarities in their combined profiles, whereas the more isolated positions of ‘Pietros’, ‘Parmen auriu’, and the ‘Caslere’–‘Mustoase’ group indicate distinctive combinations of the analyzed characteristics. These results complement the correlation and ordination analyses and further demonstrate the considerable variability present within the old apple germplasm.

### 3.2. Comprehensive Characterization of Superior Cultivars Selected for Further Investigation

**Figure 8:**
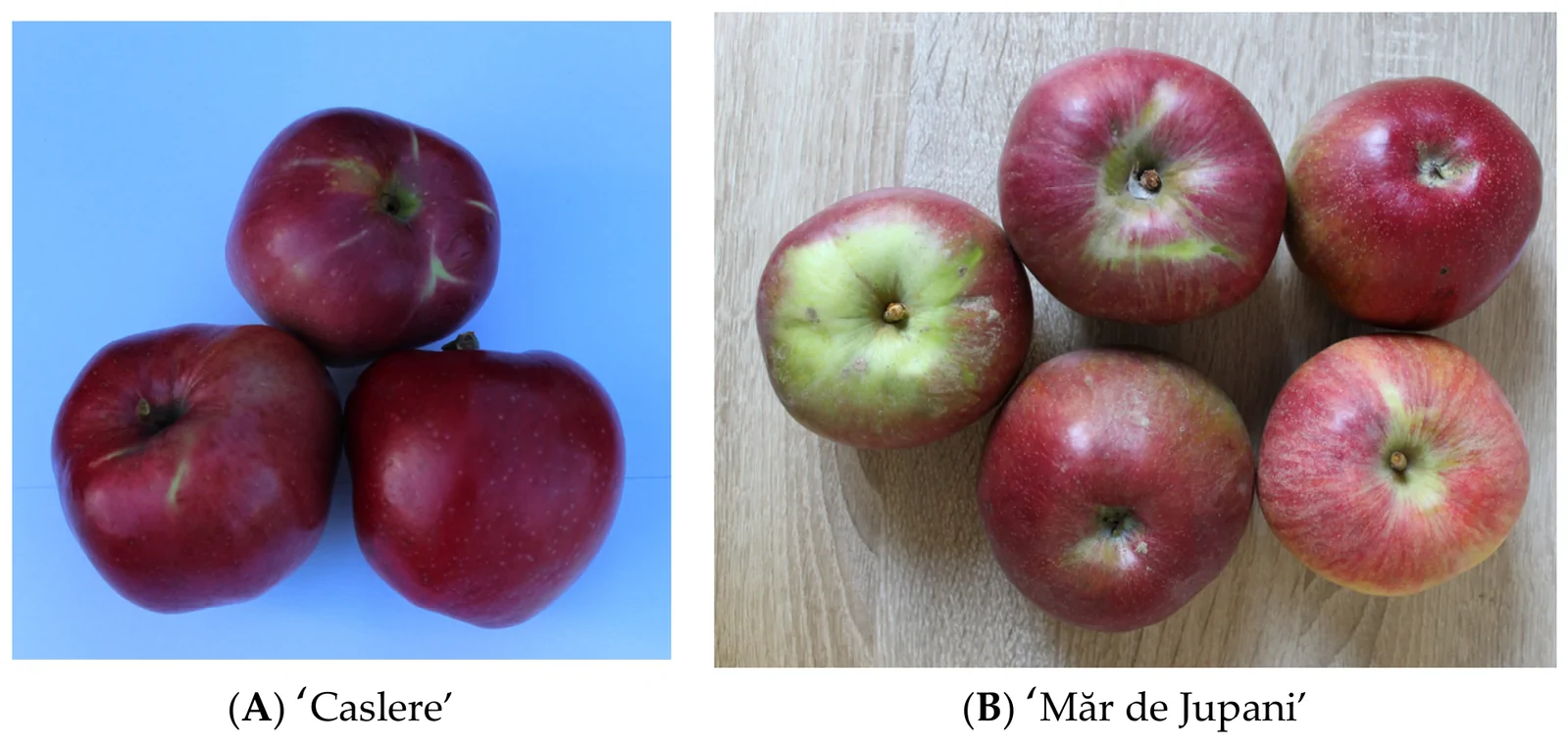
External appearance of the selected traditional apple cultivars: (**A**) ‘Caslere’ and (**B**) ‘Măr de Jupani’.

Based on the results obtained for total phenolic content, total flavonoid content, and antioxidant activity, two cultivars exhibiting distinct and superior phytochemical characteristics, ‘Caslere’ and ‘Măr de Jupani’, were selected for further characterization of their individual phenolic profiles and colour parameters (Figure 8).

#### 3.2.1. Individual Polyphenolic Profile

Rosmarinic acid was the main phenolic compound in the flesh of both cultivars, with a significantly higher concentration in ‘Măr de Jupani’ (153.60 µg/mL) compared with ‘Caslere’ (66.17 µg/mL). In both cultivars, resveratrol and quercetin were identified as the next most abundant phenolic compounds, following the same distribution pattern, with higher concentrations in the flesh of ‘Măr de Jupani’ (56.95 µg/mL and 52.84 µg/mL, respectively) than in ‘Caslere’ (27.84 µg/mL and 14.81 µg/mL, respectively) (Table 7).

The peel exhibited distinct phenolic profiles between the two cultivars. In ‘Măr de Jupani’ rosmarinic acid was the predominant phenolic compound (125.42 µg/mL), whereas epicatechin was the major constituent in ‘Caslere’ (62.35 µg/mL). In both cultivars, resveratrol was among the major phenolic constituents, reaching concentrations of 41.36 and 36.47 µg/mL in ‘Măr de Jupani’ and ‘Caslere’, respectively (Table 8).

#### 3.2.2. Colour Analysis

‘Caslere’ cultivar exhibited a more than 2-fold-higher a* value in the peel (Table 9), indicating a more intense red coloration, which was associated with a higher total flavonoid content (Table 4). HPLC analysis revealed greater concentrations of epicatechin and quercetin in the peel of ‘Caslere’ compred with ‘Măr de Jupani’ (Table 8). This observation is consistent with the reported association between red peel pigmentation and flavonoid accumulation in apples [74,75]. In addition, ‘Caslere’ cultivar showed lower L* values in both peel and flesh, reflecting a darker appearance.

‘Măr de Jupani’ cultivar exhibited higher L* and b* values in the flesh (Table 9), indicating a lighter and more yellow coloration. This cultivar also showed higher concentrations of rosmarinic acid, resveratrol, and quercetin in the flesh, although no direct relationship between these phenolic compounds and flesh colour can be deduced from the present results.

## 4. Discussion

Total phenolic and flavonoid contents, as well as antioxidant activity, have generally been reported to be higher in traditional and local apple cultivars compared with modern cultivars [76,77,78]. The present study confirmed the high phytochemical potential of traditional apple cultivars from western Romania, revealing their value as sources of bioactive compounds and supporting the nutritional benefits associated with the consumption of whole fruits.

Phenolic compounds are recognized as the primary contributors to the antioxidant activity of apples [68,79], as ascorbic acid accounts for less than 0.4% of the total antioxidant activity [80]. Their antioxidant properties are mainly attributed to the ability of phenolic hydroxyl groups to donate electrons or hydrogen atoms, thereby neutralizing reactive oxygen species.

The polyphenolic profile of apples is strongly influenced by cultivar, genetic background, fruit tissue, geographical area, soil, climatic conditions, harvest period, storage and processing conditions [51,81,82,83,84,85,86]. Studies of traditional and ancient apple cultivars from Italy, Croatia and Portugal have likewise reported considerable variation in phenolic composition and concentration among cultivars and between peel and flesh. Italian studies have highlighted the high phenolic content and antioxidant capacity of several traditional cultivars [47,87,88], while Croatian and Portuguese cultivars have also shown marked cultivar- and tissue-dependent differences in polyphenolic accumulation [15,89,90]. These findings, together with the present results, suggest that phenolic accumulation is determined by the interaction between cultivar-specific genetic characteristics and environmental conditions. Differences in climate, light exposure, temperature and water availability may contribute to variation in phenolic biosynthesis among geographically distinct apple populations, although methodological differences among studies should also be considered when comparing absolute values. In the present study, these factors were reflected in the considerable variation observed among the investigated traditional cultivars, both in total phenolic and flavonoid contents and in their distribution between peel and flesh.

In the present study, these factors were reflected in the considerable variation observed among the investigated traditional cultivars, both in total phenolic and flavonoid contents and in their distribution between peel and flesh.

Using the Folin–Ciocâlteu method, total phenolic content in 18 local apple cultivars ranged from 857.87 ± 7.21 µg gallic acid equivalents (GAE)/g dry matter (DM) in ’Parmen auriu’ to 2361.18 ± 31.86 µg GAE/g DM in ‘Măr de Jupani’. The variability observed among cultivars highlights the substantial influence of genetic background on phenolic accumulation, even among apples originating from the same geographical region. These values are generally consistent with those reported in the literature [91], although variations among the studies are expected due to differences in genotype, environmental conditions, fruit maturity, and analytical procedures. In particular, the inclusion or exclusion of seeds, which contain high concentrations of phenolic compounds, may substantially influence the reported values.

As the distribution of phenolic compounds varies among different tissues of the fruits [67], separate analyses were performed for peel and flesh samples. Polyphenol content in apple flesh ranged from 323.54 ± 4.73 µg GAE/g DM (‘Parmen auriu’) to 1111.65 ± 16.03 µg GAE/g DM (‘Aore’), values that are comparable with previously reported data [51,62]. Although similar cultivar-dependent trends have been reported previously, the concentrations of polyphenols observed in lyophilized flesh samples were substantially higher, reflecting methodological differences and sample preparation: ‘Pasalma’ (12.55 mg GAE/g; 12,550 μg GAE/g), ‘Tetovka’ (11.45 mg GAE/g; 11,450 μg GAE/g), ‘Livadarka’ (11.22 mg GAE/g; 11,220 μg GAE/g), ‘Golden Delicious’ (10.40 mg GAE/g; 10,400 μg GAE/g) and ‘Belle de Boskoop’ (10.08 mg GAE/g; 10,080 μg GAE/g). The superior phenolic content of autochtonous cultivars has also been reported [92].

Apple peel is widely recognized as the fruit tissue richest in bioactive compounds, including phenolic compounds, carotenoids and anthocyanins [83,93,94]. This enrichment contributes to the superior antioxidant capacity of peel tissues. In agreement with previous studies, the peel of the cultivars investigated in our study exhibited significantly higher polyphenol concentrations than the flesh. On average, an approximately 1.9-fold higher phenolic content was identified in the peel, a value consistent with the 1.2–3.3-fold differences reported by Drogoudi et al. [83]. Comparable peel-associated high phenolic content has been shown across several apple cultivars [51,53,62,90,92,95].

The peel’s biological function as the main defense against external stressors, such as UV radiation, infections, and mechanical damage, explains its high phenolic concentration. As a result, secondary metabolites that have protective and antioxidant properties tend to build up in epidermal tissues.

Flavonoid analysis revealed a similar pattern. Total flavonoid contents ranged from 3908.3 ± 10.79 to 7306.2 ± 86.29 µg rutin equivalents (RE)/g DM, values comparable with those reported for traditional cultivars from other regions [96,97,98]. According to earlier studies, flavonoid concentrations in apple peel are typically three to six times higher than in the flesh [18,53,68]. The predominance of flavonoids in the peel observed in the present study is therefore consistent with their protective role in outer fruit tissues.

Since phenolic compounds are major contributors to antioxidant capacity, the variation observed in their accumulation among cultivars was expected to be reflected in antioxidant activity.

Antioxidant activity is strongly correlated with polyphenolic content and can vary depending on cultivar, fruit tissue, fruit maturity, and environmental conditions [65,82,99,100]. Results of the present study indicated that phenolic compounds were predominantly concentrated in the epidermal tissue and were primarily responsible for the free radical scavenging activity observed in the fruit extracts [101]. Flesh extracts exhibited inhibition values ranging from 44.41 ± 0.39% to 88.88 ± 0.05% (mean: 75.89%), whereas in peel extracts inhibition percentage ranged from 33.55 ± 0.02% to 99.51 ± 0.02% (mean: 84.86%). The nearly 10% higher mean inhibition observed in the peel corresponded to a 1.77-fold stronger bioactive effect.

Similarly, the mean half-maximal inhibitory concentration (IC_50_) value for peel extracts (2.74 mg/mL) was substantially lower than the one recorded for flesh extracts (4.73 mg/mL), confirming the superior antioxidant activity of the peel. These findings are consistent with numerous previous reports indicating that antioxidant activity in apple peel may be 1.5–9.2 times higher than in the flesh [67,83,102]. The results also support earlier observations that local cultivars possess stronger antioxidant activity than imported commercial cultivars, as reflected by their lower IC_50_ values [98].

Pearson correlation analysis confirmed the contribution of phenolic compounds to the antioxidant potential of apple tissues, with the strongest relationships observed in peel extracts. Significant negative correlations between total polyphenol content/total flavonoid content (TPC/TFC) and IC_50_ values demonstrated that cultivars with higher phenolic accumulation exhibited stronger antioxidant activity, while the positive correlations with percentage inhibition further supported their role in free radical scavenging activity. The stronger associations detected in the peel, particularly for flavonoid content, are consistent with the preferential localization of phenolic compounds in epidermal tissues, where they contribute both to fruit pigmentation and protection against oxidative stress. In contrast, the weaker correlations detected in flesh extracts suggest that antioxidant capacity is influenced not only by the total concentration of phenolic compounds but also by the qualitative composition of individual phenolics. Overall, these findings highlight the role of phenolic compounds, especially flavonoids, in determining the biofunctional properties of apples. Given the association between oxidative stress and the development of chronic diseases, including hypertension, cancer, hyperlipidemia, diabetes, cardiovascular and neurodegenerative disorders, consumption of apples rich in polyphenols may contribute to disease prevention and health promotion [103,104,105].

To gain deeper insight into qualitative differences in phenolic composition, two cultivars with contrasting phytochemical characteristics (‘Caslere’ and ‘Măr de Jupani’) were selected for High-Performance Liquid Chromatography (HPLC) and colour analysis. The two cultivars exhibited distinct phenolic profiles. Rosmarinic acid and resveratrol were among the predominant phenolic compounds in both cultivars, whereas epicatechin was particularly abundant in the peel of ‘Caslere’ cultivar. These differences indicate cultivar- and tissue-dependent variation in the accumulation of individual phenolic compounds.

Rosmarinic acid has previously been identified in apple tissues, although its concentration varies considerably depending on cultivar, tissue type and analytical approach [106,107,108]. The cultivar-dependent accumulation of rosmarinic acid in apples suggests that its content may be influenced by differences in phenylpropanoid metabolism and by environmental factors affecting secondary metabolite biosynthesis [5,109,110]. In the present study, ‘Măr de Jupani’ cultivar exhibited a distinctive phenolic profile, characterized by the predominance of rosmarinic acid in both peel (125.42 µg/mL) and flesh (153.60 µg/mL). The consistent accumulation of this compound across fruit tissues, together with its markedly higher concentrations compared with ‘Caslere’, suggests a cultivar-specific pattern of phenolic accumulation.

Resveratrol, a phenolic compound widely reported in plants, including apples, has been associated with plant defense responses to biotic and abiotic stresses [111,112]. It exhibits a broad spectrum of biological activities, including free radical scavenging and antioxidant effects [113]. In the present study, resveratrol was among the predominant phenolic compounds, although its concentration varied between tissues and cultivars, further supporting the contribution of traditional apples to dietary intake of bioactive stilbenes [114].

Epicatechin is considered one of the major phenolic compounds in apples, particularly in the peel, where flavan-3-ols contribute to the formation of proanthocyanidins [110,115,116]. In agreement with previous studies [102,117], the present results showed a higher accumulation of epicatechin in the peel of ‘Caslere’ cultivar (62.35 µg/mL), indicating cultivar-related differences in flavonoid composition. Such variability has been attributed primarily to genetic factors influencing phenolic biosynthesis and accumulation [81,83].

These cultivar-specific phenolic profiles may contribute to differences in antioxidant activity, as the antioxidant capacity of apples is strongly influenced not only by the total amount of phenolic compounds but also by their qualitative composition. Interestingly, the high phenolic content observed in ‘Caslere’ is consistent with its previously reported moderate tolerance to several major apple diseases and pests, including apple scab, monilia, codling moth, green apple aphid and leaf-curling aphid [54]. Although phenolic compounds are known to participate in plant defense responses, this co-occurrence does not establish a causal relationship between phenolic accumulation and disease or pest tolerance, as these traits were not evaluated concurrently in the present study. For ‘Măr de Jupani’, which exhibited the highest total phenolic content, comparable information on disease or drought tolerance was not available.

The two cultivars exhibited distinct color profiles, with ‘Caslere’ showing darker and more intensely red-colored peel, as indicated by lower L* and higher a* values, whereas ‘Măr de Jupani’ presented lighter tissues and a more pronounced yellow colour in the flesh, reflected by higher L* and b* values.

The more intense redness observed in the peel of ‘Caslere’ was associated with increased flavonoid accumulation and higher levels of specific flavonoids, including epicatechin and quercetin. This indicates a relationship between peel coloration and flavonoid composition.

In contrast, the lighter coloration of ‘Măr de Jupani’ was accompanied by a different phenolic profile, characterized by the predominant accumulation of rosmarinic acid in both peel and flesh. These results indicate that differences in peel coloration are not necessarily associated by an overall increase in all phenolic compounds, as cultivars may differ in the preferential accumulation of specific phenolic classes.

The relationship observed in the present study is consistent with previous reports showing that peel colour represents an important indicator of phytochemical composition. Red-skinned apples generally exhibit higher antioxidant activity than green-skinned cultivars due to the accumulation of anthocyanins, particularly cyanidin 3-O-galactoside [118,119,120]. Research comparing cultivars with varying peel colors has repeatedly shown that red apples have higher anthocyanin contents and antioxidant activity than yellow or green ones [93,121].

Comparative analyses have demonstrated that commercial cultivars often contain lower polyphenol concentrations than traditional or local cultivars [17,92,122,123,124,125]. This trend may be partially explained by consumer preferences for sweeter, more palatable apples with reduced susceptibility to enzymatic browning. Polyphenols serve as substrates for polyphenol oxidase (PPO), which catalyzes oxidative browning reactions; consequently, higher polyphenol contents are frequently associated with more intense browning. Consequently, current breeding programmes often prioritize texture, sweetness, shelf life and reduced browning susceptibility over phenolic content [126,127]. This could help to explain why traditional cultivars typically have higher concentrations of phenolic compounds than commercial ones.

The variability in phenolic content given by genetic factors has significant implications for both breeding programs and conservation initiatives, because traditional cultivars may serve as major reservoirs of health-promoting phytochemicals that are progressively being lost due to the replacement of local germplasm by commercial cultivars.

The results of the current study have significant practical applications. Since apple peel contains substantially higher concentrations of bioactive compounds than the flesh, consumption of whole fruits may increase dietary exposure to these compounds. However, their nutritional relevance should also be considered in terms of bioaccessibility. In vitro and ex vivo digestion study has shown that approximately 63–82% of phenolic compounds were released from apple flesh during the oral phase of digestion, compared with 42–58% from the peel, with bioaccessibility varying among phenolic groups [126]. Thus, the higher phenolic concentration of the peel does not necessarily translate proportionally into bioavailability. Despite this difference, the higher concentration and greater diversity of phenolics in the peel support the consumption of fresh apples with the peel, while thorough chewing may facilitate their release during digestion.

Beyond the direct consumption of fresh apples, the valorization of apple-processing by-products represents an attractive strategy for increasing the utilization of peel-derived bioactive compounds.

Apple pomace consists mainly of peel and pulp (approximately 95%), along with seeds (2–4%) and stems (around 1%) [128]. Globally, apple juice production generates an estimated 4–5 million tons of pomace annually, yet only a limited proportion is commercially utilized [129,130], primarily in bakery items and apple cider vinegar. The remaining fraction is often discarded in open fields, or accumulated near juice-processing facilities, creating serious environmental concerns [130,131]. Given its high concentration of bioactive compounds, apple pomace is recognized for its potential health-promoting effects [128,132,133,134]. The food industry should adopt appropriate recovery and valorization strategies to efficiently extract these bioactive compounds and incorporate them as functional ingredients into value-added products.

From a valorization perspective, apple pomace represents a valuable source of phenolic compounds, pectin, dietary fibre, minerals and other bioactive constituents with potential applications as functional food ingredients [135]. Sustainable extraction approaches, including ultrasound-assisted extraction (UAE), microwave-assisted extraction (MAE) and thermal-stirred extraction (TSE), have been successfully applied to recover valuable compounds from apple pomace. A comparative study using water as the extraction solvent demonstrated that all three techniques could effectively recover polyphenols from apple pomace, with optimal extraction achieved after 10 min for TSE and UAE and only 5 min for MAE. Among the investigated techniques, MAE showed the most favourable overall performance, combining high polyphenol extraction efficiency with the lowest environmental impact, while the use of water as a solvent further reduced the environmental impacts of all three methods [136]. Phenolic-rich extracts and pomace-derived ingredients have also been incorporated into functional food products, including probiotic-containing fermented products, baked and extruded products, and fruit bars, enhancing their phenolic content and antioxidant properties [137,138,139,140].

Overall, these approaches support the conversion of apple pomace into value-added ingredients that could be explored for use in functional beverages and as natural antioxidant, binding or coating agents in food products. Such valorization is consistent with contemporary concepts of sustainable food production and circular economy, converting an underutilized by-product into a source of both nutritional and economical value [140].

## 5. Conclusions

This study demonstrated the significant biochemical diversity of 18 traditional apple cultivars from western Romania. The main findings of the study can be summarized as follows:

Total phenolic content ranged from 857.87 to 2361.18 μg gallic acid equivalents (GAE)/g dry matter (DM), while total flavonoid content ranged from 3908.30 to 7306.20 μg rutin equivalents (RE)/g DM.

Apple peel was generally richer in bioactive compounds than the flesh, confirming the importance of peel as a major source of these compounds. However, ‘Aore’ and ‘Pietros’ showed distinct flesh-dominant accumulation patterns, highlighting the cultivar-dependent distribution of bioactive constituents.

Peel generally exhibited stronger antioxidant activity than flesh. The mean half-maximal inhibitory concentration (IC_50_) was 2.74 mg/mL for peel and 4.73 mg/mL for flesh, while particularly strong antioxidant activity was observed in the peels of ‘Caslere’ and ‘Măr de Jupani’. ‘Aore’ showed the strongest antioxidant activity among flesh extracts.

Among the investigated cultivars, ‘Caslere’ and ‘Măr de Jupani’ appeared to be particularly rich in phenolic compounds, further supported by their distinct individual phenolic compositions identified through High-Performance Liquid Chromatography (HPLC) analysis and differences in colour characteristics.

Beyond their nutritional value, the preservation and promotion of such traditional cultivars contribute to biodiversity conservation and cultural heritage. Cultivars with high phenolic content hold particular promise for health-oriented applications, and their targeted cultivation and consumption could enhance dietary antioxidant intake. Future research should investigate the impact of processing and storage on the stability and bioaccessibility of these bioactive compounds to support their optimal use in the food industry.

A limitation of the present study is that antioxidant activity was assessed using only the 2,2-diphenyl-1-picrylhydrazyl (DPPH) assay, while comprehensive phenolic profiling was performed only for ‘Caslere’ and ‘Măr de Jupani’cultivars. It should also be noted that the analyses were based on a single batch per cultivar; therefore, potential within-cultivar biological variability could not be assessed. Future studies should therefore extend phenolic characterization to all cultivars using techniques such as High-Performance Liquid Chromatography- Mass Spectrometry (HPLC-MS) and employ complementary antioxidant assays to provide a more comprehensive assessment of antioxidant potential. In vivo studies would further help determine the biological relevance of the observed antioxidant activity, while optimization of cultivation conditions and harvest timing could be investigated as strategies to enhance phenolic biosynthesis and the functional value of apple fruit and its by-products.

## Figures and Tables

**Table 1:** Soluble solid content (SSC) for harvested fruits (°Brix).

Apple Cultivar	SSC (^°^Brix)
‘Aore’	11.6
‘Botu Oii Alb’	15.8
‘Botu Oii de Caraș’	17.0
‘Carigate’	15.4
‘Caslere’	18.56
‘Domnesc’	13.2
‘Jonathan de munte’	17.9
‘Jonathan’	18.16
‘Mustoase’	15.8
‘Măr de Jupani’	16.0
‘Parmen auriu’	18.26
‘Pietros’	14.53
‘Pogace’	16.66
‘Pătul de Vârciorova’	15.2
‘Pătul’	15.26
‘Șovare’	17.73
‘Țigănesc’	13.13
‘Vițate’	20.03

**Table 2:** Concentrations carried out for each extract.

Dilution Series	Extract Concentration (µL/mL)	Final Volume (mL)
1	250	1
2	400	1
3	500	1
4	750	1
5	900	1

**Table 3:** Fruit morphometric characteristics of the investigated apple cultivars. Values are expressed as means.

Cultivar	Small Diameter (mm)	Large Diameter (mm)	Height (mm)	Mass (g)	Fruit Shape Index (FSI)
‘Aore’	55.31	77.08	59.46	175.52	0.77
‘Botu Oii Alb’	43.61	57.81	61.80	98.87	1.06
‘Botu Oii de Caraș’	46.91	65.84	58.60	121.67	0.89
‘Carigate’	62.37	77.23	56.44	153.63	0.73
‘Caslere’	58.45	83.85	71.33	239.16	0.85
‘Domnesc’	51.78	72.12	65.55	150.53	0.90
‘Jonathan de munte’	57.67	76.97	59.97	171.46	0.77
‘Jonathan’	68.41	77.66	58.94	168.15	0.75
‘Mustoase’	69.11	91.60	67.69	254.53	0.73
‘Măr de Jupani’	55.08	75.75	58.91	162.83	0.77
‘Parmen auriu’	59.69	79.71	64.74	192.66	0.81
‘Pietros’	57.22	78.17	60.42	166.63	0.77
‘Pogace’	56.42	79.72	64.37	190.03	0.80
‘Pătul de Vârciorova’	64.78	73.62	60.22	172.5	0.81
‘Pătul’	59.26	78.25	59.84	184.54	0.76
‘Șovare’	59.77	76.44	62.17	181.67	0.81
‘Țigănesc’	56.51	76.47	68.36	178.25	0.89
‘Vițate’	53.25	68.65	63.99	132.65	0.93

**Table 4:** Phenolic content of flesh and peel of the investigated apple cultivars. Values are expressed as means (µg GAE/g DM) ± SD (n = 3).

Cultivar	Phenolic Content of Flesh (µg GAE/g DM)	Phenolic Content of Peel (µg GAE/g DM)	Total Phenolic Content (µg GAE/g DM)
‘Aore’	1111.65 ± 16.03 bE *	574.74 ± 6.68 aBC	1686.40 ± 9.36
‘Botu Oii Alb’	484.0 ± 7.58 aAB	584.86 ± 8.04 bBC	1068.87 ± 0.98
‘Botu Oii de Caraș’	414.54 ± 2.63 aAB	857.56 ± 9.76 bCDE	1272.10 ± 12.39
‘Carigate’	441.75 ± 11.74 aAB	754.0 ± 10.76 bBCDE	1195.75 ± 21.91
‘Caslere’	479.09 ± 8.39 aAB	1474.47 ± 78.44 bG	1953.57 ± 86.29
‘Domnesc ’	749.67 ± 10.80 aCD	885.58 ± 4.61 bDE	1635.26 ± 7.41
‘Jonathan de munte’	361.34 ± 5.40 aA	894.72 ± 13.11 bDE	1256.07 ± 8.06
‘Jonathan’	382.0 ± 10.71 aA	964.01 ± 17.78 bEF	1346.01 ± 7.06
‘Mustoase’	498.02 ± 4.00 aAB	492.59 ± 1.16 aB	990.61 ± 2.88
‘Măr de Jupani’	863.83 ± 4.87 aD	1497.35 ± 27.08 bG	2361.18 ± 31.86
‘Parmen auriu’	323.54 ± 4.73 aA	534.33 ± 2.60 bB	857.87 ± 7.21
‘Pietros’	689.55 ± 7.59 aC	218.17 ± 3.48 bA	907.73 ± 10.79
‘Pogace’	438.08 ± 3.63 aAB	1166.74 ± 15.99 bF	1604.83 ± 13.05
‘Pătul de Vârciorova’	481.97 ± 13.41 aAB	706.21 ± 3.08 bBCDE	1188.18 ± 10.43
‘Pătul’	588.10 ± 7.35 aBC	943.71 ± 8.54 bEF	1531.81 ± 4.65
‘Șovare’	340.09 ± 4.94 aA	639.85 ± 3.79 bBCD	979.94 ± 8.18
‘Țigănesc’	375.84 ± 1.19 aA	686.04 ± 10.87 bBCDE	1061.89 ± 10.11
‘Vițate’	397.85 ± 2.48 aA	616.14 ± 4.69 bBCD	1013.99 ± 3.97

* Different lowercase letters indicate statistically significant differences between the phenolic content of flesh and peel, while different capital letters indicate statistically significant differences between cultivars within flesh and peel respectively, according to Tukey’s HSD test at *p* < 0.05.

**Table 5:** Flavonoid content of flesh and peel of the investigated apple cultivars. Values are expressed as mean *(*µg RE/g DM) ± SD (n = 3).

Cultivar	Flavonoid Content of Flesh (µg RE/g DM)	Flavonoid Content of Peel (µg RE/g DM)	Total Flavonoid Content (µg RE/g DM)
‘Aore’	3422.70 ± 2.26 bH *	2626.03 ± 25.41 aBC	6048.74 ± 9.36
‘Botu Oii Alb’	2414.36 ± 0.97 aDEFG	2990.03 ± 2.74 bBCD	5404.4 ± 0.98
‘Botu Oii de Caraș’	2039.62 ± 1.04 aBCDE	3973.52 ± 1.80 bFG	6013.16 ± 12.39
‘Carigate’	1507.33 ± 1.84 aAB	3746.0 ± 2.97 bEFG	5253.34 ± 21.91
‘Caslere’	2510.14 ± 1.37 aEFG	4796.05 ± 0.37 bH	7306.20 ± 86.29
‘Domnesc’	2932.67 ± 6.96 aG	3859.64 ± 6.90 bEFG	6792.31 ± 7.41
‘Jonathan de munte’	1629.15 ± 0.40 aABC	3882.82 ± 1.41 bEFG	5511.97 ± 8.06
‘Jonathan’	1875.09 ± 1.04 aABCD	4188.56 ± 2.94 bGH	6063.66 ± 7.06
‘Mustoase’	2357.64 ± 1.73 aDEF	2370.76 ± 1.49 aB	4728.40 ± 2.88
‘Măr de Jupani’	3697.16 ± 0.36 aH	3522.84 ± 2.52 aDEFG	7220.01 ± 31.86
‘Parmen auriu’	1426.01 ± 0.61 aA	2488.25 ± 32.05 bB	3914.27 ± 7.21
‘Pietros’	2750.85 ± 0.86 bFG	1157.43 ± 1.90 aA	3908.30 ± 10.79
‘Pogace’	1645.37 ± 1.38 aABC	3330.13 ± 1.58 bCDEF	4975.51 ± 13.05
‘Pătul de Vârciorova’	2060.48 ± 7.40 aBCDE	2826.52 ± 4.21 bBCD	4887.01 ± 10.43
‘Pătul’	2105.62 ± 5.06 aCDE	3387.51 ± 8.20 bDEF	5493.14 ± 4.65
‘Șovare’	1619.40 ± 2.85 aABC	2970.23 ± 2.68 bBCD	4589.63 ± 8.18
‘Țigănesc’	1634.55 ± 1.72 aABC	3205.09 ± 0.18 bCDE	4839.64 ± 10.11
‘Vițate’	1934.80 ± 0.65 aABCD	3248.83 ± 1.23 bCDE	5183.63 ± 3.97

* Different lowercase letters indicate statistically significant differences between the phenolic content of flesh and peel, while different capital letters indicate statistically significant differences between cultivars within flesh and peel respectively, according to Tukey’s HSD test at *p* < 0.05.

**Table 6:** The IC_50_ and percentage inhibitions of flesh and peel of the studied apple cultivars.

Cultivar	IC_50_ (mg/mL) Flesh	Percentage Inhibition (%) Flesh	IC_50_ (mg/mL) Peel	Percentage Inhibition (%) Peel
‘Aore’	1.93 ± 0.0262 aA *	88.86 ± 0.0314 h	3.48 ± 0.0077 fgA	88.73 ± 0.1458 d
‘Botu Oii Alb’	4.84 ± 0.0129 efghA	77.75 ± 0.2047 ef	2.92 ± 0.0085 defA	88.67 ± 0.0809 de
‘Botu Oii de Caraș’	3.75 ± 0.0036 bcdA	88.82 ± 0.1332 gh	2.16 ± 0.0073 abcdeA	88.87 ± 0.0120 de
‘Carigate’	5.52 ± 0.0149 ghA	66.69 ± 0.2308 d	2.82 ± 0.0083 defA	66.39 ± 0.0139 b
‘Caslere’	4.89 ± 0.0658 efghB	88.80 ± 9.099 fg	1.11 ± 0.0020 aA	99.51 ± 0.0199 e
‘Domnesc’	3.00 ± 0.0076 abA	88.88 ± 0.0481 h	2.20 ± 0.0023 abcdeA	88.39 ± 0.0108 de
‘Jonathan de munte’	5.09 ± 0.0595 fghB	66.65 ± 0.2790 cd	1.91 ± 0.0065 abcdA	88.19 ± 0.0139 de
‘Jonathan’	6.61 ± 0.0586 ijB	55.56 ± 0.1915 b	2.38 ± 0.0420 bcdefA	88.67 ± 0.1635 de
‘Mustoase’	5.07 ± 0.0137 fghB	66.69 ± 0.1005 d	1.76 ± 0.0040 abcdA	88.46 ± 0.0709 de
‘Măr de Jupani’	5.33 ± 0.3262 ghB	66.69 ± 0.6122 d	1.19 ± 0.0039 abA	99.31 ± 2.8542 e
‘Parmen auriu’	7.42 ± 0.0246 jA	44.41 ± 0.3931 a	4.66 ± 0.0300 gA	77.56 ± 0.1021 c
‘Pietros’	3.67 ± 0.0115 bcA	88.84 ± 0.1094 gh	7.22 ± 0.0021 hB	33.55 ± 0.0192 a
‘Pogace’	5.84 ± 0.0219 hiB	66.61 ± 0.2240 bc	1.43 ± 0.0022 abcA	88.19 ± 0.0155 de
‘Pătul de Vârciorova’	4.67 ± 0.0203 cdefgA	88.84 ± 0.0410 gh	3.34 ± 0.0137 efA	88.87 ± 0.1318 de
‘Pătul’	4.25 ± 0.0195 cdefA	77.76 ± 0.3442 ef	2.43 ± 0.0022 cdefA	88.97 ± 0.0623 de
‘Șovare’	4.79 ± 0.0075 defghA	77.78 ± 0.1346 fg	2.64 ± 0.0099 cdefA	88.46 ± 0.1515 de
‘Țigănesc’	4.71 ± 0.0173 cdefgA	77.70 ± 0.2003 de	3.42 ± 0.0162 fA	88.53 ± 0.0970 d
‘Vițate’	3.90 ± 0.0089 bcdeA	88.83 ± 0.1640 gh	2.38 ± 0.0054 bcdefA	88.28 ± 0.0785 de

* The values presented are means ± SD (n = 3). Different lowercase letters indicate significant differences between apple cultivars within the same parameter, while different capital letters indicate significant differences between flesh and peel.

**Table 7:** High-Performance Liquid Chromatography (HPLC)-determined individual polyphenolic profile of apple flesh from the two selected cultivars.

Phenolic Compound	‘Caslere’ (µg/mL)	‘Măr de Jupani’ (µg/mL)
Gallic acid	UD	UD
Epicatechin	9.392	UD
Caffeic acid	UD	UD
Beta-Resorcylic acid	UD	UD
Coumaric acid	UD	11.676
Ferulic acid	4.107	7.014
Rosmarinic acid	66.176	153.609
Resveratrol	27.840	56.958
Quercetin	14.812	52.844

UD: undetectable.

**Table 8:** HPLC-determined individual polyphenolic profile of apple peel from the two selected cultivars.

Phenolic Compound	‘Caslere’ (µg/mL)	‘Măr de Jupani’ (µg/mL)
Gallic acid	UD	UD
Epicatechin	62.354	UD
Caffeic acid	26.636	UD
Beta-Resorcylic acid	UD	11.166
Coumaric acid	4.108	4.925
Ferulic acid	6.251	19.740
Rosmarinic acid	32.527	125.421
Resveratrol	36.474	41.362
Quercetin	23.127	6.886

UD: undetectable.

**Table 9:** Peel and flesh color parameters of the two selected cultivars. Values are expressed as means ± SD (n = 3).

Cultivar	Peel L*	Peel a*	Peel b*	Flesh L*	Flesh a*	Flesh b*
‘Caslere’	42.34	13.73	23.22	53.05	13.73	23.22
‘Măr de Jupani’	58.35	5.74	23.2	62.01 ± 3.43	12.38 ± 2.52	31.63 ± 0.83

## Data Availability

The original contributions presented in this study are included in the article. Further inquiries can be directed to the corresponding author.

## Abbreviations

The following abbreviations are used in this manuscript:

UV	Ultraviolet
PPO	Polyphenol oxidase
GAE	Gallic acid equivalents
DM	Dry matter
SD	Standard deviation
RE	Rutin equivalents
DPPH	2,2-diphenyl-1-picrylhydrazyl
IC_50_	Half-maximal inhibitory concentration
HPLC	High-Performance Liquid Chromatography
FSI	Fruit shape index
ANOVA	One-way analysis of variance
PCA	Principal component analysis
CA	Correspondence analysis
HCA	Hierarchical cluster analysis
TPC	Total polyphenol content
MdLAR1	Leucoanthocyanidin Reductase 1
TFC	Total flavonoid content
UAE	Ultrasound-assisted extraction
MAE	Microwave-assisted extraction
TSE	Thermal-stirred extraction
HPLC-MS	High-Performance Liquid Chromatography- Mass Spectrometry

## References

[1] Fotirić Akšić M., Mutić J., Tešić Ž., Meland M. (2020). Evaluation of fruit mineral contents of two apple cultivars grown in organic and integrated production systems. Acta Hortic..

[2] Vallée Marcotte B., Verheyde M., Pomerleau S., Doyen A., Couillard C. (2022). Health Benefits of Apple Juice Consumption: A Review of Interventional Trials on Humans. Nutrients.

[3] Behl T., Kumar K., Brisc C., Rus M., Nistor-Cseppento D.C., Bustea C., Corb Aron R.A., Pantis C., Zengin G., Sehgal A. (2021). Exploring the multifocal role of phytochemicals as immunomodulators. Biomed. Pharmacother..

[4] Mierczak K., Garus-Pakowska A. (2024). An Overview of Apple Varieties and the Importance of Apple Consumption in the Prevention of Non-Communicable Diseases—A Narrative Review. Nutrients.

[5] Francini A., Sebastiani L. (2013). Phenolic Compounds in Apple (*Malus x domestica* Borkh): Compounds Characterization and Stability during Postharvest and after Processing. Antioxidants.

[6] Haminiuk C.W.I., Maciel G.M., Plata-Oviedo M.S.V., Peralta R.M. (2012). Phenolic compounds in fruits—An overview. Int. J. Food Sci. Technol..

[7] da Silva L.C., Viganó J., de Souza Mesquita L.M., Baião Dias A.L., de Souza M.C., Sanches V.L., Chaves J.O., Pizani R.S., Contieri L.S., Rostagno M.A. (2021). Recent advances and trends in extraction techniques to recover polyphenols compounds from apple by-products. Food Chem. X.

[8] Kalinowska M., Gryko K., Wróblewska A.M., Jabłońska-Trypuć A., Karpowicz D. (2020). Phenolic content, chemical composition and anti-/pro-oxidant activity of Gold Milenium and Papierowka apple peel extracts. Sci. Rep..

[9] Acquavia M.A., Pascale R., Foti L., Carlucci G., Scrano L., Martelli G., Brienza M., Coviello D., Bianco G., Lelario F. (2021). Analytical Methods for Extraction and Identification of Primary and Secondary Metabolites of Apple (*Malus domestica*) Fruits: A Review. Separations.

[10] Kalinowska M., Bielawska A., Lewandowska-Siwkiewicz H., Priebe W., Lewandowski W. (2014). Apples: Content of phenolic compounds vs. variety, part of apple and cultivation model, extraction of phenolic compounds, biological properties. Plant Physiol. Biochem..

[11] Hyson D.A. (2011). A Comprehensive Review of Apples and Apple Components and Their Relationship to Human Health. Adv. Nutr..

[12] Ahmed S., Stepp J. (2016). Beyond yields: Climate change effects on specialty crop quality and agroecological management. Elementa.

[13] Arnold M., Gramza-Michalowska A. (2024). Recent Development on the Chemical Composition and Phenolic Extraction Methods of Apple (*Malus domestica*)—A Review. Food Bioproc. Tech..

[14] Boyer J., Liu R.H. (2004). Apple phytochemicals and their health benefits. Nutr. J..

[15] Jakobek L., Barron A.R. (2016). Ancient apple varieties from Croatia as a source of bioactive polyphenolic compounds. J. Food Compos. Anal..

[16] Li M.-J., Ma F.-W., Zhang M., Pu F. (2008). Distribution and metabolism of ascorbic acid in apple fruits (*Malus domestica* Borkh cv. Gala). Plant Sci..

[17] Kschonsek J., Wolfram T., Stöckl A., Böhm V. (2018). Polyphenolic Compounds Analysis of Old and New Apple Cultivars and Contribution of Polyphenolic Profile to the In Vitro Antioxidant Capacity. Antioxidants.

[18] Kunradi Vieira F.G., Borges G.S.C., Copetti C., Gonzaga L.V., Nunes E.C., Fett R. (2009). Activity and contents of polyphenolic antioxidants in the whole fruit, flesh and peel of three apple cultivars. Arch. Latinoam. Nutr..

[19] Lavic D., Radovic M., Aliman J., Badzak N., Kulina M., Hadziabulic A., Ilhan G., Mureșan C., Marc R.A. (2023). Influence of cultivar and fertilization treatment on bioactive content of some apple (*Malus domestica* Borkh.) cultivars. Turk. J. Agric..

[20] Sun S., Liu Z., Lin M., Gao N., Wang X. (2024). Polyphenols in health and food processing: Antibacterial, anti-inflammatory, and antioxidant insights. Front. Nutr..

[21] Ferrario G., Baron G., Gado F., Della Vedova L., Bombardelli E., Carini M., D’Amato A., Aldini G., Altomare A. (2022). Polyphenols from Thinned Young Apples: HPLC-HRMS Profile and Evaluation of Their Anti-Oxidant and Anti-Inflammatory Activities by Proteomic Studies. Antioxidants.

[22] Saxena S., Verma J., Gautam S. (2016). Potential Prophylactic Properties of Apple and Characterization of Potent Bioactive from cv. “Granny Smith” Displaying Strong Antimutagenicity in Models Including Human Lymphoblast TK6+/− Cell Line. J. Food Sci..

[23] Gautam S., Saxena S., Kumar S. (2016). Fruits and Vegetables as Dietary Sources of Antimutagens. J. Food Chem. Nanotechnol..

[24] Tu S.-H., Chen L.-C., Ho Y.-S. (2017). An apple a day to prevent cancer formation: Reducing cancer risk with flavonoids. J. Food Drug Anal..

[25] Patocka J., Bhardwaj K., Klimova B., Nepovimova E., Wu Q., Landi M., Kuca K., Valis M., Wu W. (2020). *Malus domestica*: A Review on Nutritional Features, Chemical Composition, Traditional and Medicinal Value. Plants.

[26] Saarenhovi M., Salo P., Scheinin M., Lehto J., Lovró Z., Tiihonen K., Lehtinen M.J., Junnila J., Hasselwander O., Tarpila A. (2017). The effect of an apple polyphenol extract rich in epicatechin and flavan-3-ol oligomers on brachial artery flow-mediated vasodilatory function in volunteers with elevated blood pressure. Nutr. J..

[27] Iqbal I., Wilairatana P., Saqib F., Nasir B., Wahid M., Latif M.F., Iqbal A., Naz R., Mubarak M.S. (2023). Plant Polyphenols and Their Potential Benefits on Cardiovascular Health: A Review. Molecules.

[28] Guo X.-F., Yang B., Tang J., Jiang J.-J., Li D. (2017). Apple and pear consumption and type 2 diabetes mellitus risk: A meta-analysis of prospective cohort studies. Food Funct..

[29] Thilakarathna S.H., Rupasinghe H.V., Needs P.W. (2013). Apple peel bioactive rich extracts effectively inhibit in vitro human LDL cholesterol oxidation. Food Chem..

[30] Rubido S., Garcia-Caballero L., Abeleira M.T., Limeres J., Garcia M., Diz P. (2018). Effect of chewing an apple on dental plaque removal and on salivary bacterial viability. PLoS ONE.

[31] Carrasco-Pozo C., Speisky H., Brunser O., Pastene E., Gotteland M. (2011). Apple Peel Polyphenols Protect against Gastrointestinal Mucosa Alterations Induced by Indomethacin in Rats. J. Agric. Food Chem..

[32] Pastene E., Speisky H., García A., Moreno J., Troncoso M., Figueroa G. (2010). In Vitro and in Vivo Effects of Apple Peel Polyphenols against Helicobacter pylori. J. Agric. Food Chem..

[33] Piccolo E.L., Landi M., Massai R., Remorini D., Conte G., Guidi L. (2019). Ancient apple cultivars from Garfagnana (Tuscany, Italy): A potential source for ‘nutrafruit’ production. Food Chem..

[34] Oszmiański J., Lachowicz S., Gamsjäger H. (2020). Phytochemical analysis by liquid chromatography of ten old apple varieties grown in Austria and their antioxidative activity. Eur. Food Res. Technol..

[35] Wang X., Li C., Liang D., Zou Y., Li P., Ma F. (2015). Phenolic compounds and antioxidant activity in red-fleshed apples. J. Funct. Foods.

[36] Sethi S., Joshi A., Arora B., Bhowmik A., Sharma R.R., Kumar P. (2020). Significance of FRAP, DPPH, and CUPRAC assays for antioxidant activity determination in apple fruit extracts. Eur. Food Res. Technol..

[37] Shen N., Wang T., Gan Q., Liu S., Wang L., Jin B. (2022). Plant flavonoids: Classification, distribution, biosynthesis, and antioxidant activity. Food Chem..

[38] Maleki S.J., Crespo J.F., Cabanillas B. (2019). Anti-inflammatory effects of flavonoids. Food Chem..

[39] Can Z., Dincer B., Sahin H., Baltas N., Yildiz O., Kolayli S. (2013). Polyphenol oxidase activity and antioxidant properties of Yomra apple (*Malus communis* L.) from Turkey. J. Enzym. Inhib. Med. Chem..

[40] Boeckx T., Winters A., Webb K.J., Kingston-Smith A.H. (2017). Detection of Potential Chloroplastic Substrates for Polyphenol Oxidase Suggests a Role in Undamaged Leaves. Front. Plant Sci..

[41] Akagic A., Oras A., İmrak B., Kafkas N.E.Y. (2025). Traditional versus Commercial Apple Varieties: Chemical Composition and Implications for Processing. Malus domestica—New Insights.

[42] Prichko T., Ulyanovskaya E., Droficheva N. (2020). Evaluation of biochemical indicators of apple fruits quality for the complex selection of the valuable source material for breeding. BIO Web Conf..

[43] Feliciano R.P., Antunes C., Ramos A., Serra A.T., Figueira M.E., Duarte C.M.M., Carvalho A.d., Bronze M.R. (2010). Characterization of traditional and exotic apple varieties from Portugal. Part 1—Nutritional, phytochemical and sensory evaluation. J. Funct. Foods.

[44] Militaru M., Butac M., Sumedrea M., Călinescu M., Marin F. (2015). Evaluation of resistance to pests and diseases of some old apple varieties. Fruit Grow. Res..

[45] Gasi F., Simon S., Pojskic N., Kurtovic M., Pejic I. (2010). Genetic assessment of apple germplasm in Bosnia and Herzegovina using microsatellite and morphologic markers. Sci. Hortic..

[46] Alihodzic A., Gasi F., Drkenda P., Akagic A., Oras A., Meland M., Music O., Spaho N. (2018). Sensory Acceptability of the Autochthonous Fruits of Bosnia and Herzegovina: Challenges and Possibilities for Food Industry. Erwerbs-Obstbau.

[47] Preti R., Tarola A.M. (2021). Study of polyphenols, antioxidant capacity and minerals for the valorisation of ancient apple cultivars from Northeast Italy. Eur. Food Res. Technol..

[48] Ponte L.G.S., Pavan I.C.B., Mancini M.C.S., da Silva L.G.S., Morelli A.P., Severino M.B., Bezerra R.M.N., Simabuco F.M. (2021). The Hallmarks of Flavonoids in Cancer. Molecules.

[49] Rana S., Bhushan S. (2016). Apple phenolics as nutraceuticals: Assessment, analysis and application. J. Food Sci. Technol..

[50] Bai X., Zhang H., Ren S. (2013). Antioxidant activity and HPLC analysis of polyphenol-enriched extracts from industrial apple pomace. J. Sci. Food Agric..

[51] Khanizadeh S., Tsao R., Rekika D., Yang R., Charles M.T., Vasantha Rupasinghe H.P. (2008). Polyphenol composition and total antioxidant capacity of selected apple genotypes for processing. J. Food Compos. Anal..

[52] Biedrzycka E., Amarowicz R. (2008). Diet and Health: Apple Polyphenols as Antioxidants. Food Rev. Int..

[53] Kim I., Ku K., Jeong M., Kim S.S., Mitchell A., Lee J. (2019). A comparison of the chemical composition and antioxidant activity of several new early- to mid-season apple cultivars for a warmer climate with traditional cultivars. J. Sci. Food Agric..

[54] Iordănescu O.A., Radulov I., Dascălu I., Berbecea A., Camen D., Orboi M.D., Călin C.C., Gal T.E. (2023). Comparative Study on the Behavior of Some Old Apple Varieties before and after Their Grafting, with Potential for Use in Urban Horticulture. Horticulturae.

[55] Tripon R., Repone C., Țurai M., Chiș C., Oiegaș S., Boldura O.M., Tulcan C. (2020). Performance Characteristic Evaluation of Folin-Ciocalteu Micro-Method for Total Polyphenols Determination from Plant Extracts. Lucr. Ştiinţ. Med. Vet..

[56] Al-Matani S.K., Al-Wahabi R.N.S., Hossain M.A. (2015). Total flavonoids content and antimicrobial activity of crude extract from leaves of Ficus sycomorus native to Sultanate of Oman. Karbala Int. J. Mod. Sci..

[57] Protocol de Screening al Eficientei Antiradicalice a Fito-Compusilor Utilizand DPPH. https://proiecte.incdsb.ro/fitoplat/wp-content/uploads/2017/09/Protocol-1.3.pdf.

[58] Cho K., Choi E.-S., Kim J.-H., Son J.-W., Kim E. (2022). Numerical learning of deep features from drug-exposed cell images to calculate IC50 without staining. Sci. Rep..

[59] Dossa S., Neagu C., Lalescu D., Negrea M., Stoin D., Jianu C., Berbecea A., Cseh L., Rivis A., Suba M. (2025). Evaluation of the Nutritional, Rheological, Functional, and Sensory Properties of Cookies Enriched with Taro (*Colocasia esculenta*) Flour as a Partial Substitute for Wheat Flour. Foods.

[60] Рэнцэндава Ч., Székely D., Furulyás D., Végvári G., Gonelimali F., Kumar P., M S.-M. (2020). Stability of Carotene and Phenols of Sea Buckthorn (*Hippophae rhamnoides* L.) Juice with Pomace during Storage. Period. Polytech. Chem. Eng..

[61] Lang L., Fujimura K., Dujmovic N., Gray S., Knaap E. (2006). Development of a Controlled Vocabulary and Software Application to Analyze Fruit Shape Variation in Tomato and Other Plant Species. Plant Physiol..

[62] Khanizadeh S., Tsao R., Rekika D., Yang R., DeEll J. (2007). Phenolic composition and antioxidant activity of selected apple genotypes. J. Food Agric. Environ..

[63] Raudone L., Raudonis R., Liaudanskas M., Janulis V., Viskelis P. (2017). Phenolic antioxidant profiles in the whole fruit, flesh and peel of apple cultivars grown in Lithuania. Sci. Hort..

[64] Petkovsek M.M., Stampar F., Veberic R. (2007). Parameters of inner quality of the apple scab resistant and susceptible apple cultivars (*Malus domestica* Borkh.). Sci. Hort..

[65] Valavanidis A., Vlachogianni T., Psomas A., Zovoili A., Siatis V. (2009). Polyphenolic profile and antioxidant activity of five apple cultivars grown under organic and conventional agricultural practices. Int. J. Food Sci. Technol..

[66] Khan S.A., Chibon P.-Y., de Vos R.C.H., Schipper B.A., Walraven E., Beekwilder J., van Dijk T., Finkers R., Visser R.G.F., van de Weg E.W. (2012). Genetic analysis of metabolites in apple fruits indicates an mQTL hotspot for phenolic compounds on linkage group 16. J. Exp. Bot..

[67] Manzoor M., Anwar F., Saari N., Ashraf M. (2012). Variations of Antioxidant Characteristics and Mineral Contents in Pulp and Peel of Different Apple (*Malus domestica* Borkh.) Cultivars from Pakistan. Molecules.

[68] Tsao R., Yang R., Xie S., Sockovie E., Khanizadeh S. (2005). Which Polyphenolic Compounds Contribute to the Total Antioxidant Activities of Apple?. J. Agric. Food Chem..

[69] Butkeviciute A., Abukauskas V., Janulis V., Kviklys D. (2022). Phenolic Content and Antioxidant Activity in Apples of the ‘Galaval’ Cultivar Grown on 17 Different Rootstocks. Antioxidants.

[70] Stagos D. (2020). Antioxidant Activity of Polyphenolic Plant Extracts. Antioxidants.

[71] Kunradi Vieira F.G., Borges G.S.C., Copetti C., Di Pietro P.F., Nunes E.C., Fett R. (2011). Phenolic compounds and antioxidant activity of the apple flesh and peel of eleven cultivars grown in Brazil. Sci. Hort..

[72] Yuri J.A., Neira A., Quilodran A., Motomura Y., Palomo I. (2009). Antioxidant activity and total phenolics concentration in apple peel and flesh is determined by cultivar and agroclimatic growing regions in Chile. J. Food Agric. Environ..

[73] Łata B. (2008). Apple peel antioxidant status in relation to genotype, storage type and time. Sci. Hort..

[74] Lister C.E., Lancaster J.E., Walker J.R.L. (1996). Developmental Changes in Enzymes of Flavonoid Biosynthesis in the Skins of Red and Green Apple Cultivars. J. Sci. Food Agric..

[75] Bu H., Gu G., Hu Y., Yang Y., Yang L., Yuan H., Yu W. (2025). Research Advances in the Synthesis and Regulation of Apple Anthocyanins. Biology.

[76] Balik J., Rop O., Mlček J., Híc P., Horák M., Řezníček V. (2012). Assessment of nutritional parameters of native apple cultivars as new gene sources. Acta Univ. Agric. Silvic. Mendel. Brun..

[77] Donno D., Loris B., Mellano M.G., Torello Marinoni D., Kim C., Sara C., Giancarlo B. (2012). Application of sensory, nutraceutical and genetic techniques to create a quality profile of ancient apple cultivars. J. Food Qual..

[78] Minnocci A., P I., Martinelli F., Sebastiani L. (2010). Micromorphological, biochemical, and genetic characterization of two ancient, late-bearing apple varieties. Eur. J. Hortic. Sci..

[79] Lee K.W., Kim Y.J., Kim D.-O., Lee H.J., Lee C.Y. (2003). Major Phenolics in Apple and Their Contribution to the Total Antioxidant Capacity. J. Agric. Food Chem..

[80] Eberhardt M.V., Lee C.Y., Liu R.H. (2000). Antioxidant activity of fresh apples. Nature.

[81] McGhie T.K., Hunt M., Barnett L.E. (2005). Cultivar and Growing Region Determine the Antioxidant Polyphenolic Concentration and Composition of Apples Grown in New Zealand. J. Agric. Food Chem..

[82] Scalzo J., Politi A., Pellegrini N., Mezzetti B., Battino M. (2005). Plant genotype affects total antioxidant capacity and phenolic contents in fruit. Nutrition.

[83] Drogoudi P.D., Michailidis Z., Pantelidis G. (2008). Peel and flesh antioxidant content and harvest quality characteristics of seven apple cultivars. Sci. Hortic..

[84] Henríquez C., Speisky H., Chiffelle I., Valenzuela T., Araya M., Simpson R., Almonacid S. (2010). Development of an ingredient containing apple peel, as a source of polyphenols and dietary fiber. J. Food Sci..

[85] Kunradi Vieira F.G., Di Pietro P.F., da Silva E.L., Borges G.S.C., Nunes E.C., Fett R. (2012). Improvement of serum antioxidant status in humans after the acute intake of apple juices. Nutr. Res..

[86] Dweck A.C. (2009). The internal and external use of medicinal plants. Clin. Dermatol..

[87] Ceci A.T., Bassi M., Guerra W., Oberhuber M., Robatscher P., Mattivi F., Franceschi P. (2021). Metabolomic Characterization of Commercial, Old, and Red-Fleshed Apple Varieties. Metabolites.

[88] Panzella L., Petriccione M., Rega P., Scortichini M., Napolitano A. (2013). A reappraisal of traditional apple cultivars from Southern Italy as a rich source of phenols with superior antioxidant activity. Food Chem..

[89] Duralija B., Putnik P., Brdar D., Bebek Markovinović A., Zavadlav S., Pateiro M., Domínguez R., Lorenzo J.M., Bursać Kovačević D. (2021). The Perspective of Croatian Old Apple Cultivars in Extensive Farming for the Production of Functional Foods. Foods.

[90] Teixeira J.D., Soares Mateus A.R., Sanchez C., Parpot P., Almeida C., Sanches Silva A. (2023). Antioxidant Capacity and Phenolics Profile of Portuguese Traditional Cultivars of Apples and Pears and Their By-Products: On the Way to Newer Applications. Foods.

[91] Montero L., Herrero M., Ibanez E., Cifuentes A. (2013). Profiling of phenolic compounds from different apple varieties using comprehensive two-dimensional liquid chromatography. J. Chromatogr. A.

[92] Petkovska A., Gjamovski V., Stefova M. (2016). Comparison of different extraction solvents for assay of the polyphenol content in the peel and pulp of apple cultivars from Macedonia. Maced. J. Chem. Chem. Eng..

[93] Michailidis M., Karagiannis E., Nasiopoulou E., Skodra C., Molassiotis A., Tanou G. (2021). Peach, Apple, and Pear Fruit Quality: To Peel or Not to Peel?. Horticulturae.

[94] Delgado-Pelayo R., Gallardo-Guerrero L., Hornero-Méndez D. (2014). Chlorophyll and carotenoid pigments in the peel and flesh of commercial apple fruit varieties. Food Res. Int..

[95] Ran J., Sun H., Xu Y., Wang T., Zhao R. (2016). Comparison of Antioxidant Activities and High-PerformanceLiquid Chromatography Analysis of Polyphenol from Different Apple Varieties. Int. J. Food Prop..

[96] Liaudanskas M., Viškelis P., Jakštas V., Raudonis R., Kviklys D., Milašius A., Janulis V. (2014). Application of an Optimized HPLC Method for the Detection of Various Phenolic Compounds in Apples from Lithuanian Cultivars. J. Chem..

[97] Liaudanskas M., Zymone K., Viškelis J., Janulis V. (2018). Optimisation of the extraction of flavonoids from apples using response surface methodology. Ital. J. Food Sci..

[98] Samid I., Khouya T., Elbouny H., Sellam K., Alem C. (2023). Quantification of phytochemical and antioxidant properties of aqueous apple extracts from the high atlas mountains in Morocco and their anti-inflammatory effect on Wistar rats. Sci. Afr..

[99] Łata B. (2007). Relationship between Apple Peel and the Whole Fruit Antioxidant Content:  Year and Cultivar Variation. J. Agric. Food Chem..

[100] Matthes A., Schmitz-Eiberger M. (2009). Polyphenol content and antioxidant capacity of apple fruit: Effect of cultivar and storage conditions. J. Appl. Bot. Food Qual..

[101] D’Abrosca B., Pacifico S., Cefarelli G., Mastellone C., Fiorentino A. (2007). ‘Limoncella’ apple, an Italian apple cultivar: Phenolic and flavonoid contents and antioxidant activity. Food Chem..

[102] Chinnici F., Bendini A., Gaiani A., Riponi C. (2004). Radical Scavenging Activities of Peels and Pulps from cv. Golden Delicious Apples as Related to Their Phenolic Composition. J. Agric. Food Chem..

[103] Wu K.H., Ho C.T., Chen Z.F., Chen L.C., Whang-Peng J., Lin T.N., Ho Y.S. (2018). The apple polyphenol phloretin inhibits breast cancer cell migration and proliferation via inhibition of signals by type 2 glucose transporter. J. Food Drug Anal..

[104] Goh B.H., Tan J.B.L., Swamy M.K. (2020). Cardiovascular Benefits of Dietary Polyphenols. Plant-Derived Bioactives: Production, Properties and Therapeutic Applications.

[105] Birben E., Sahiner U.M., Sackesen C., Erzurum S., Kalayci O. (2012). Oxidative Stress and Antioxidant Defense. World Allergy Organ. J..

[106] Hossain M., Salehuddin S.M., Mizanur R.S.M., Kabir M.J. (2010). The effect of solvents on recovery of polyphenols from the pink fuji apple skin. Afr. J. Food Agric. Nutr. Dev..

[107] Lee J., Chan B.L., Mitchell A.E. (2017). Identification/quantification of free and bound phenolic acids in peel and pulp of apples (*Malus domestica*) using high resolution mass spectrometry (HRMS). Food Chem..

[108] Veberic R., Trobec M., Herbinger K., Hofer M., Grill D., Stampar F. (2005). Phenolic compounds in some apple (*Malus domestica* Bork) cultivars of organic and integrated production. J. Sci. Food Agric..

[109] McClure K.A., Gong Y., Song J., Vinqvist-Tymchuk M., Campbell Palmer L., Fan L., Burgher-MacLellan K., Zhang Z., Celton J.M., Forney C.F. (2019). Genome-wide association studies in apple reveal loci of large effect controlling apple polyphenols. Hortic. Res..

[110] Li Y., Sun H., Li J., Qin S., Niu Z., Qiao X., Yang B. (2021). Influence of genetic background, growth latitude and bagging treatment on phenolic compounds in fruits of commercial cultivars and wild types of apples (*Malus* sp.). Eur. Food Res. Technol..

[111] Salehi B., Mishra A.P., Nigam M., Sener B., Kilic M., Sharifi-Rad M., Fokou P.V.T., Martins N., Sharifi-Rad J. (2018). Resveratrol: A Double-Edged Sword in Health Benefits. Biomedicines.

[112] Zheng X., Chen H., Su Q., Wang C., Sha G., Ma C., Sun Z., Yang X., Li X., Tian Y. (2021). Resveratrol improves the iron deficiency adaptation of *Malus baccata* seedlings by regulating iron absorption. BMC Plant Biol..

[113] Malhotra A., Bath S., Elbarbry F. (2015). An Organ System Approach to Explore the Antioxidative, Anti-Inflammatory, and Cytoprotective Actions of Resveratrol. Oxidative Med. Cell. Longev..

[114] Rühmann S., Treutter D., Fritsche S., Briviba K., Szankowski I. (2006). Piceid (resveratrol glucoside) synthesis in stilbene synthase transgenic apple fruit. J. Agric. Food Chem..

[115] Lister C.E., Lancaster J.E., Sutton K.H., Walker J.R.L. (1994). Developmental changes in the concentration and composition of flavonoids in skin of a red and a green apple cultivar. J. Sci. Food Agric..

[116] Henry-Kirk R.A., McGhie T.K., Andre C.M., Hellens R.P., Allan A.C. (2012). Transcriptional analysis of apple fruit proanthocyanidin biosynthesis. J. Exp. Bot..

[117] Cano-Lou J., Millán-Laleona A., Candrea R., Les F., Pina A., Caprioli G., López V. (2025). Apple peels as an edible source of phenolic bioactive compounds with antidiabetic and antiglycation properties. Food Funct..

[118] Piagentini A.M., Pirovani M.E. (2017). Total Phenolics Content, Antioxidant Capacity, Physicochemical Attributes, and Browning Susceptibility of Different Apple Cultivars for Minimal Processing. Int. J. Fruit Sci..

[119] Ding R., Che X., Shen Z., Zhang Y. (2021). Metabolome and transcriptome profiling provide insights into green apple peel reveals light- and UV-B-responsive pathway in anthocyanins accumulation. BMC Plant Biol..

[120] González-Talice J., Yuri J.A., del Pozo A. (2013). Relations among pigments, color and phenolic concentrations in the peel of two Gala apple strains according to canopy position and light environment. Sci. Hort..

[121] Wolfe K., Wu X., Liu R.H. (2003). Antioxidant Activity of Apple Peels. J. Agric. Food Chem..

[122] Vrhovsek U., Rigo A., Tonon D., Mattivi F. (2004). Quantitation of Polyphenols in Different Apple Varieties. J. Agric. Food Chem..

[123] Volz R.K., McGhie T.K. (2011). Genetic variability in apple fruit polyphenol composition in *Malus × domestica* and *Malus sieversii* germplasm grown in New Zealand. J. Agric. Food Chem..

[124] Mihailović N.R., Mihailović V.B., Kreft S., Ćirić A.R., Joksović L.G., Đurđević P.T. (2018). Analysis of phenolics in the peel and pulp of wild apples (*Malus sylvestris* (L.) Mill.). J. Food Compos. Anal..

[125] Stojiljković D., Arsić I., Tadić V. (2016). Extracts of wild apple fruit (*Malus sylvestris* (L.) Mill., Rosaceae) as a source of antioxidant substances for use in production of nutraceuticals and cosmeceuticals. Ind. Crops Prod..

[126] Kaeswurm J.A.H., Burandt M.R., Mayer P.S., Straub L.V., Buchweitz M. (2022). Bioaccessibility of Apple Polyphenols from Peel and Flesh during Oral Digestion. J. Agric. Food Chem..

[127] Moon K.M., Kwon E.-B., Lee B., Kim C.Y. (2020). Recent Trends in Controlling the Enzymatic Browning of Fruit and Vegetable Products. Molecules.

[128] Perussello C.A., Zhang Z., Marzocchella A., Tiwari B.K. (2017). Valorization of Apple Pomace by Extraction of Valuable Compounds. Compr. Rev. Food Sci. Food Saf..

[129] Guardia L., Suárez L., Querejeta N., Rodríguez Madrera R., Suárez B., Centeno T.A. (2019). Apple Waste: A Sustainable Source of Carbon Materials and Valuable Compounds. ACS Sustain. Chem. Eng..

[130] Shalini R., Gupta D.K. (2010). Utilization of pomace from apple processing industries: A review. J. Food Sci. Technol..

[131] Bhushan S., Kalia K., Sharma M., Singh B., Ahuja P.S. (2008). Processing of apple pomace for bioactive molecules. Crit. Rev. Biotechnol..

[132] Skinner R.C., Gigliotti J.C., Ku K.-M., Tou J.C. (2018). A comprehensive analysis of the composition, health benefits, and safety of apple pomace. Nutr. Rev..

[133] Tsoupras A., Gkika D.A., Markopoulos T., Curran R., Scallon C., Karali M., Kyzas G.Z., Mérillon J.-M., Riviere C., Lefèvre G. (2023). Apple Products (Apple Juice and Cider) and By-Products (Apple Pomace): Bioactive Compounds and Biological Properties. Natural Products in Beverages: Botany, Phytochemistry, Pharmacology and Processing.

[134] Puangpraphant S., Cuevas-Rodríguez E.-O., Oseguera-Toledo M., Hernández-Ledesma B., Martínez-Villaluenga C. (2022). Chapter 9—Anti-inflammatory and antioxidant phenolic compounds. Current Advances for Development of Functional Foods Modulating Inflammation and Oxidative Stress.

[135] Asif M., Javaid T., Razzaq Z.U., Khan M.K.I., Maan A.A., Yousaf S., Usman A., Shahid S. (2024). Sustainable utilization of apple pomace and its emerging potential for development of functional foods. Environ. Sci. Pollut. Res..

[136] Fraterrigo Garofalo S., Demichelis F., Peletti V., Picco L., Tommasi T., Fino D. (2025). Comparative study of polyphenol extraction using physical techniques and water as a solvent: A sustainable approach for the valorization of apple pomace. Environ. Sci. Pollut. Res..

[137] Vlad C.C., Păcularu-Burada B., Vasile A.M., Milea Ș.A., Bahrim G.-E., Râpeanu G., Stănciuc N. (2022). Upgrading the Functional Potential of Apple Pomace in Value-Added Ingredients with Probiotics. Antioxidants.

[138] Costa J.M., Wang W., Nakasu P.Y.S., Hu C., Forster-Carneiro T., Hallett J.P. (2025). Impacts of microwaves on the pectin extraction from apple pomace: Technological properties in structuring of hydrogels. Food Hydrocoll..

[139] Gurev A., Cesko T., Dragancea V., Ghendov-Mosanu A., Pintea A., Sturza R. (2023). Ultrasound- and Microwave-Assisted Extraction of Pectin from Apple Pomace and Its Effect on the Quality of Fruit Bars. Foods.

[140] Reis S.F., Rai D.K., Abu-Ghannam N. (2014). Apple pomace as a potential ingredient for the development of new functional foods. Int. J. Food Sci. Technol..

